# Trophoblast aging driven by IL33 deficiency elevates recurrent pregnancy loss risk through SNAP29 lactylation-mediated autophagy impairment

**DOI:** 10.1080/15548627.2026.2659946

**Published:** 2026-04-22

**Authors:** Jia-Jing Lu, Yan-Ran Sheng, Wen-Ting Hu, Yu-Kai Liu, Feng Xie, Ming-Qing Li, Xiao-Yong Zhu

**Affiliations:** a Laboratory for Reproductive Immunology, Hospital of Obstetrics and Gynecology, Shanghai Medical School, Fudan University, Shanghai, People’s Republic of China; b Department of Gynecology, Hospital of Obstetrics and Gynecology, Shanghai Medical School, Fudan University, Shanghai, People’s Republic of China; c Medical Center of Diagnosis and Treatment for Cervical and Intrauterine Diseases, Obstetrics and Gynecology Hospital of Fudan University, Shanghai, People’s Republic of China; d Department of Reproductive Immunology, the International Peace Maternity and Child Health Hospital, School of Medicine, Shanghai Key Laboratory of Embryo Original Disease, Shanghai Jiao Tong University, Shanghai, People’s Republic of China; e Shanghai Key Laboratory of Female Reproductive Endocrine Related Diseases, Hospital of Obstetrics and Gynecology, Fudan University, Shanghai, People’s Republic of China

**Keywords:** Autophagy, cell senescence, interleukin 33, lactylation, SNAP29, trophoblast

## Abstract

Emerging evidence implicates premature placental senescence as a central driver of pregnancy complications, though its underlying mechanisms remain elusive. Here, we report marked downregulation of IL33 (interleukin 33) in villi from unexplained recurrent pregnancy loss (URPL) patients, concomitant with elevated trophoblast senescence. More importantly, *il33* knockout mice exhibited placental senescence and impaired trophoblast invasion. Mechanistically, senescent trophoblasts displayed metabolic dysregulation – including enhanced glycolysis and lactate accumulation – which disrupted macroautophagic/autophagic flux and mitochondrial function. Lactate-induced lysine lactylation at residue K169 of SNAP29 (synaptosome associated protein 29) promoted its degradation, impairing macroautophagy/autophagy and trophoblast function, ultimately driving pregnancy loss. In interventional studies, senotherapies with metformin or dasatinib plus quercetin restored placental development and improved pregnancy outcomes in both IL33-deficient and inflammation-induced miscarriage models. Our findings establish the IL33-senescence-lactate axis as a critical pathway in URPL pathogenesis and support senomodulation as a therapeutic strategy.

**Abbreviations**: 2-DG: 2-deoxy-D-glucose; BafA1: bafilomycin A1; CHX: cycloheximide; CTB: cytotrophoblasts; D-gal: D-galactose; EVT: extravillous trophoblasts; HDAC: histone deacetylase; H2O2: hydrogen peroxide; IL33: Interleukin 33; LPS: lipopolysaccharide; SA-GLB1/β-gal: senescence-associated galactosidase beta 1; SASP: senescence-associated secretory phenotype; SNAP29: synaptosome associated protein 29; STB: syncytiotrophoblasts; UMAP: uniform manifold approximation and projection; URPL: unexplained recurrent pregnancy loss; VP: etoposide

## Introduction

Recurrent pregnancy loss (RPL) is a multifactorial condition characterized by the loss of two or more consecutive pregnancies, affecting approximately 1–2% of couples attempting to conceive [1,2]. Despite numerous advances in reproductive medicine, many cases of RPL remain unexplained (URPL), making it a significant challenge in both diagnosis and treatment [3,4].

During pregnancy establishment, trophoblasts are specialized cells that invade the maternal uterine wall, forming the placenta and enabling nutrient and gas exchange between the mother and fetus. In this process, trophoblast cells progressively invade the maternal spiral arteries, remodeling them into low-resistance blood vessels, thereby ensuring sufficient blood flow to the fetus through the placenta [5,6]. Dysfunction of trophoblast cells can lead to inadequate maternal blood supply, and many pregnancy-related disorders, such as recurrent miscarriage, preeclampsia, and fetal growth restriction [7,8]. The concept of cellular senescence was first proposed by Hayflick in 1961 [9]. During the senescence process, cells experience functional decline and produce a large number of pro-inflammatory and chemotactic factors, collectively known as the senescence-associated secretory phenotype (SASP) [10,11]. Studies have shown that with the increase in gestational age, senescent cells accumulate at the maternal-fetal interface, and are subsequently cleared to maintain senescence homeostasis. This process occurs throughout embryo implantation, placenta development, and continues until delivery [12]. Recently, we reported TNFSF14^+^ natural killer cells prevented spontaneous abortion by restricting leucine-mediated decidual stromal cell senescence [13]. However, the regulation of early placental senescence, its functional abnormalities, and their relationship with miscarriage remain unclear.

IL33 (interleukin 33), a cytokine belonging to the IL1 (interleukin 1) family. Accumulating evidence links IL33-ST2 signaling dysregulation to excessive inflammation, disrupted immune tolerance, and impaired phagocytosis by decidual macrophages in pregnancy loss [14–16]. However, it remains unknown whether reduced IL33 expression disrupts trophoblast invasion and placental development, exacerbating trophoblast senescence in URPL patients. Therefore, this study systematically interrogates the metabolic characteristics that underlies premature placental senescence in URPL, delineates the mechanistic contribution of IL33 to trophoblast senescence and functional impairment, and identifies tractable therapeutic targets for URPL.

## Results

### IL33 deficiency increases trophoblast senescence and the risk of miscarriage

**Figure 1.**
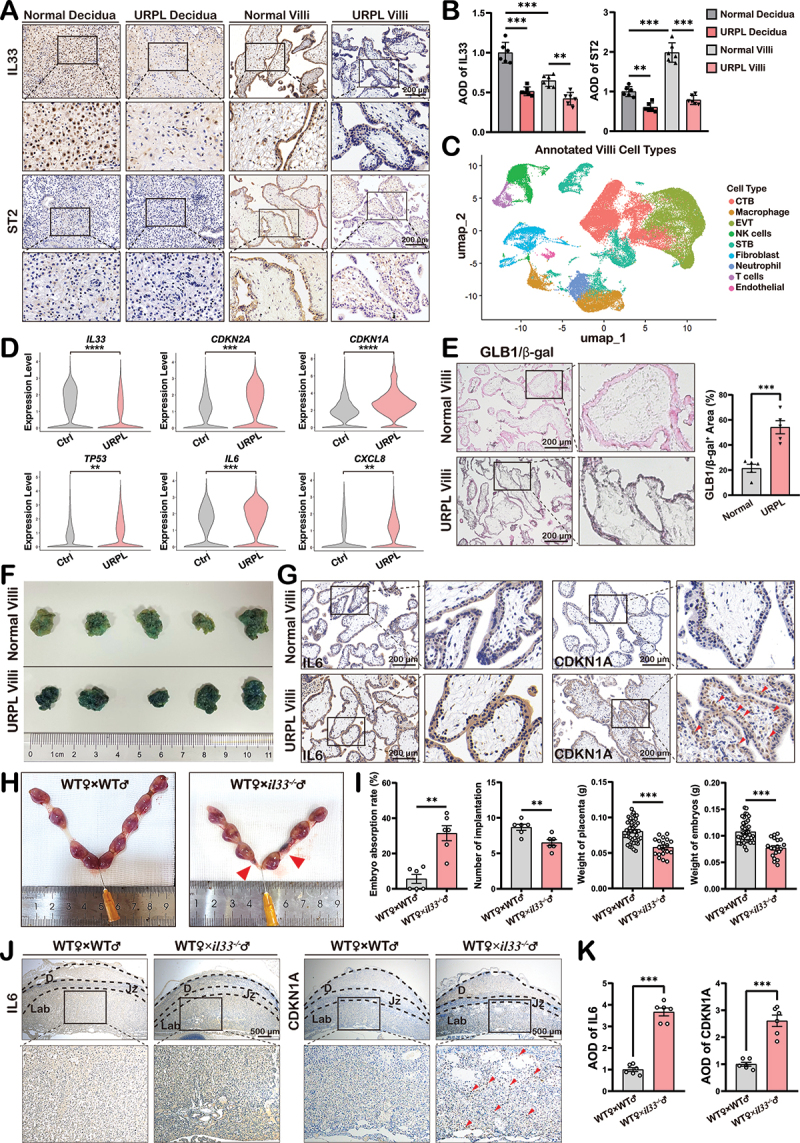
IL33 deficiency induces trophoblast senescence and leads to pregnancy loss. (**A**) The expression of IL33 and ST2 was analyzed by immunohistochemistry staining in normal or URPL patients (*n* = 6 per group). Scale bar: 200 μm. (**B**) Quantification of immunohistochemistry results. (**C**) UMAP plots of major cell types in control and URPL groups. (**D**) Violin plot showing the expression of *IL33*, *CDKN2A*, *CDKN1A*, *TP53*, *IL6* and *CXCL8* in trophoblast. (**E**) Frozen sections of normal and URPL villi were dyed with GLB1/β-gal staining (*n* = 5 per group). Scale bar: 200 μm. (**F**) Normal and URPL villous tissues at 7–9 weeks of gestation were dyed with GLB1/β-gal staining (*n* = 5 per group). (**G**) The expression of IL6 and CDKN1A/p21 was analyzed by immunohistochemistry staining in normal and URPL villi (*n* = 6 per group). Scale bar: 200 μm. (**H and I**) The pregnancy outcomes at gestational day 13.5 were assessed in wt and *il33*^−/−^ mice (*n* = 6 per group). Embryo resorption rate, number of blastocyst implantation, weight of placenta and embryo were observed in mice. (**J**) The expression of IL6 and CDKN1A was analyzed by immunohistochemistry staining in wt and *il33*^−/−^ mice (*n* = 6 per group). Scale bar: 500 μm. (**K**) Quantification of immunohistochemistry results of IL6 and CDKN1A in wt and *il33*^−/−^ mice (*n* = 6 per group). The immunohistochemistry staining was quantified with integrated optical density value. All data were generated using ImageJ. Statistical data were presented as mean ± SEM. **p < 0.01, ***p < 0.001, ****p < 0.0001, using two-tailed Student’s t-test (d, e, I, K) and one-way ANOVA test (b). [change labels to “GLB1/-gal” throughout – sorry for the previous mistake.].

Immunohistochemistry showed that IL33 was mainly expressed in the decidua and ST2 in the villi, with both significantly reduced in URPL patients (Figure 1(A,B)). A similar pattern was observed in pregnant mice (Fig. S1A, B). Subsequently, we analyzed a publicly available single-cell RNA-sequencing dataset from the NCBI Gene Expression Omnibus database, comprising villous tissues from 5 normal pregnancies and 3 URPL cases (GSE214607). After quality control and normalization, dimensionality reduction was performed using principal component analysis and uniform manifold approximation and projection (UMAP). Cells were clustered based on the shared nearest neighbor graph and annotated with canonical marker genes into nine main classes of cells: extravillous trophoblasts (EVT; expressing HLA-G, ITGA5 and MMP2), cytotrophoblasts (CTB; expressing KRT7, EGFR and TP63), syncytiotrophoblasts (STB; expressing CGA, CGB and PSG1), macrophages (expressing PTPRC, CD68 and CD14), NK cells (expressing NCAM1 and KLRD1), T cells (expressing CD3D and CD3E), fibroblast cells (expressing MME, COL1A1 and DCN), endothelial cells (expressing PECAM1, VWF and CD34), neutrophil cells (expressing S100A8, S100A9 and MPO) (Figure 1(C) and S1C). We subsequently extracted trophoblast populations (EVT, STB and CTB) to compare transcriptional profiles between two groups. In line with the immunohistochemical analysis, *IL33* expression was markedly reduced in the URPL group at the transcriptomic level. Meanwhile, the expression of several cell cycle regulators (*CDKN2A*, *CDKN1A*, *TP53*) and SASP molecules (*IL6*, *CXCL8*) was significantly elevated (Figure 1(D)), suggesting that trophoblasts in URPL patients exhibit a pronounced senescent phenotype.

To validate these findings at the tissue level, we performed senescence-associated GLB1/β-galactosidase (SA-GLB1/β-gal) staining on villous samples from 7–9 weeks of gestation. URPL villi exhibited stronger SA-GLB1/β-gal activity compared to controls (Figure 1(E,F) and S1D), along with elevated expression of IL6 and CDKN1A proteins (Figure 1(G) and S1E), further supporting the presence of premature trophoblast senescence in URPL. To further determine whether the observed trophoblast senescence and placental dysfunction were directly attributable to embryonic IL33 deficiency, we employed a paternal knockout model using *il33*^*− /−*^ male mice mated with wild-type females. Compared to controls, offsprings of *il33*^− /−^ males exhibited adverse pregnancy outcomes, including increased embryo resorption rates, reduced implantation numbers, and lower placental and fetal weights (Figure 1(H,I)). Immunohistochemical analysis of placental tissues from these offsprings revealed increased expression of IL6 and CDKN1A (Figure 1(J,K)), reinforcing the conclusion that IL33 deficiency promotes placental senescence and contributes to adverse pregnancy outcomes.

### Loss of IL33 leads to aberrant trophoblast aging and impaired placental development

**Figure 2.**
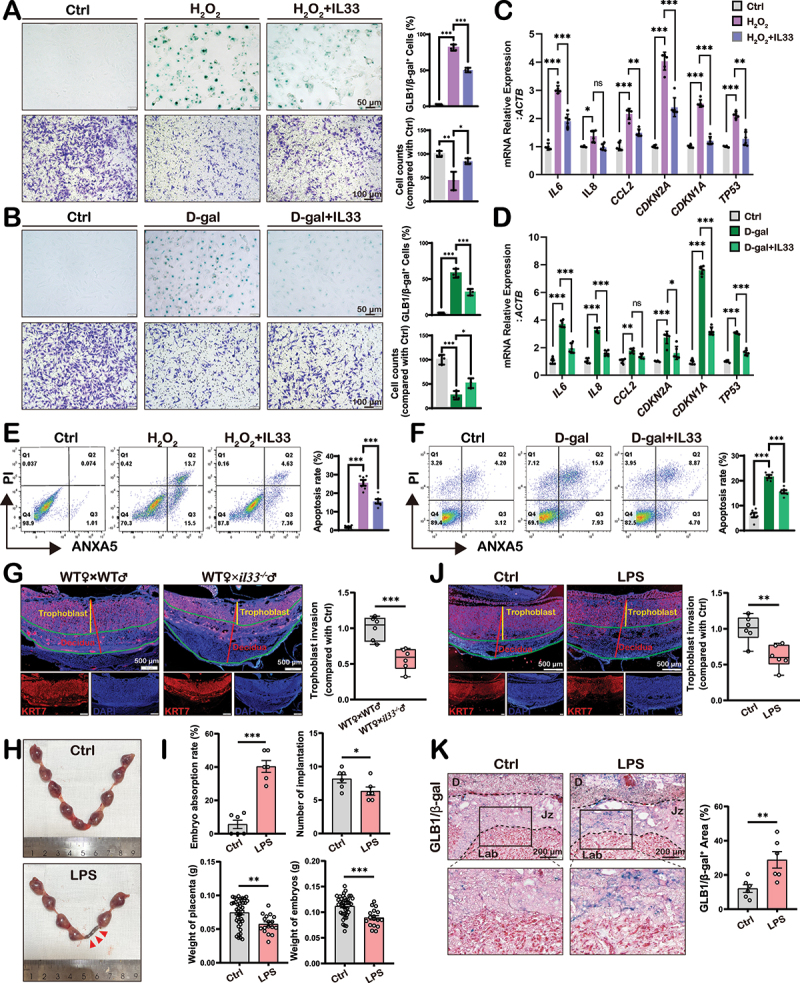
IL33 deletion triggers trophoblast premature senescence and placental developmental defects. (**A and B**) GLB1/β-galactosidase staining and Matrigel invasion assay was performed in HTR-8/SVneo cell senescence models treated with or without IL33 (10 ng/mL). Scale bar: 50 μm and 100 μm. (**C and D**) Relative mRNA expression levels of *IL6*, *IL8*, *CCL2*, *CDKN2A*, *CDKN1A* and *TP53* in HTR-8/SVneo cell senescence models treated with or without IL33 (10 ng/mL). (**E and F**) The apoptosis rate was detected by the flow cytometry assay in HTR-8/SVneo cell senescence models treated with or without IL33 (10 ng/mL). (**G**) The trophoblast infiltration depth in the placenta of wt and *il33*^−/−^ mice was measured by immunofluorescence (*n* = 6 per group). The ratio of trophoblast infiltration (yellow line) to the depth of the entire uterus (red line) was analyzed. Scale bar: 500 μm. (**H and I**) The pregnancy outcome at the gestation of day 13.5 in control and LPS-treated mice (*n* = 6 per group). Embryo resorption rate, number of blastocyst implantation, weight of placenta and embryo were observed in mice. (**J**) The trophoblast infiltration depth in the placenta of control and LPS-treated mice was measured by immunofluorescence (*n* = 6 per group). The ratio of trophoblast infiltration (yellow line) to the depth of the entire uterus (red line) was analyzed. Scale bar: 500 μm. (**K**) Placental senescence was assessed by GLB1/β-galactosidase staining in control and LPS-treated mice (*n* = 6 per group). Scale bar: 200 μm. All data were generated using ImageJ. Statistical data were presented as mean ± SEM. *p < 0.05, **p < 0.01, ***p < 0.001, ns, no significant difference, using two-tailed Student’s t-test (G-K) and one-way ANOVA test (A-F). [use italics for mRNAs.].

To explore whether exogenous supplementation of IL33 could exert anti-aging effects, we established cell senescence models in HTR-8/SVneo and JAR cells using various induction methods, including hydrogen peroxide (H_2_O_2_), D-galactose (D-gal) and etoposide (VP). As shown, senescent trophoblasts displayed a markedly reduced invasive capacity, whereas IL33 supplementation significantly decreased the number of GLB1/β-galactosidase-positive cells and enhanced their invasive ability (Figure 2(A,B) and S2A-B). Furthermore, IL33 decreased the expression of several SASP molecules (*IL6*, *IL8, CCL2*) and cell cycle regulatory proteins (*CDKN2A*, *CDKN1A*, *TP53*) (Figure 2(C,D) and S2C-D), indicating its suppressive role in trophoblast senescence. In parallel, we observed increased apoptosis in senescent trophoblasts, which was significantly ameliorated by IL33 treatment (Figure 2(E–F) and S2E-F).

Immunofluorescence analysis further revealed a reduced depth of placental invasion into maternal tissues in *il33*^−/−^ mice (Figure 2(G)), indicating that IL33 deletion impairs trophoblast invasion. Considering that inflammation is a known contributor to cellular senescence [17], we established an inflammatory model by administering lipopolysaccharide (LPS) to pregnant mice. In the LPS-treated group, embryo absorption rates were significantly increased, while both fetal and placental weights were markedly reduced (Figure 2(H,I)). Immunofluorescence further revealed diminished trophoblast infiltration into the maternal decidua, indicating impaired placental invasiveness and functional insufficiency (Figure 2(J)). Strong positive signals were showed by GLB1/β-galactosidase staining, confirming that inflammation triggered by LPS may induce premature placental senescence (Figure 2K). Moreover, qPCR analysis revealed that both il33 KO and LPS-treated placentas exhibited elevated levels of inflammatory cytokines (*Il6, Cxcl1, Tnf, Il1b, Tgfb1*), with a more pronounced increase observed in the LPS group (Fig. S2G, H), supporting the presence of an inflammatory milieu in both models. Notably, the junctional zone appeared particularly sensitive to senescence-associated changes, suggesting that it should be an early indicator region for detecting placental aging. These findings suggest that, in addition to IL33 deficiency, LPS- induced inflammation or other pathological conditions can also trigger trophoblast senescence, which represent a shared pathological pathway underlying miscarriage of diverse etiologies.

### IL33 deficiency-driven senescence disrupts autophagy in trophoblasts

Subsequently, we induced cellular senescence using H_2_O_2_, with or without exogenous IL33 supplementation. Proteomic analysis found a large number of differential proteins before and after cell senescence, most of them were located in the nucleus (Fig. S3A). GO enrichment analysis of differentially expressed proteins revealed significant changes in the activities of various desaturases, hydrolases and oxidoreductases, indicating that senescent cells exhibit abnormalities in metabolism or post-translational modifications (Fig. S3B-E).

**Figure 3.**
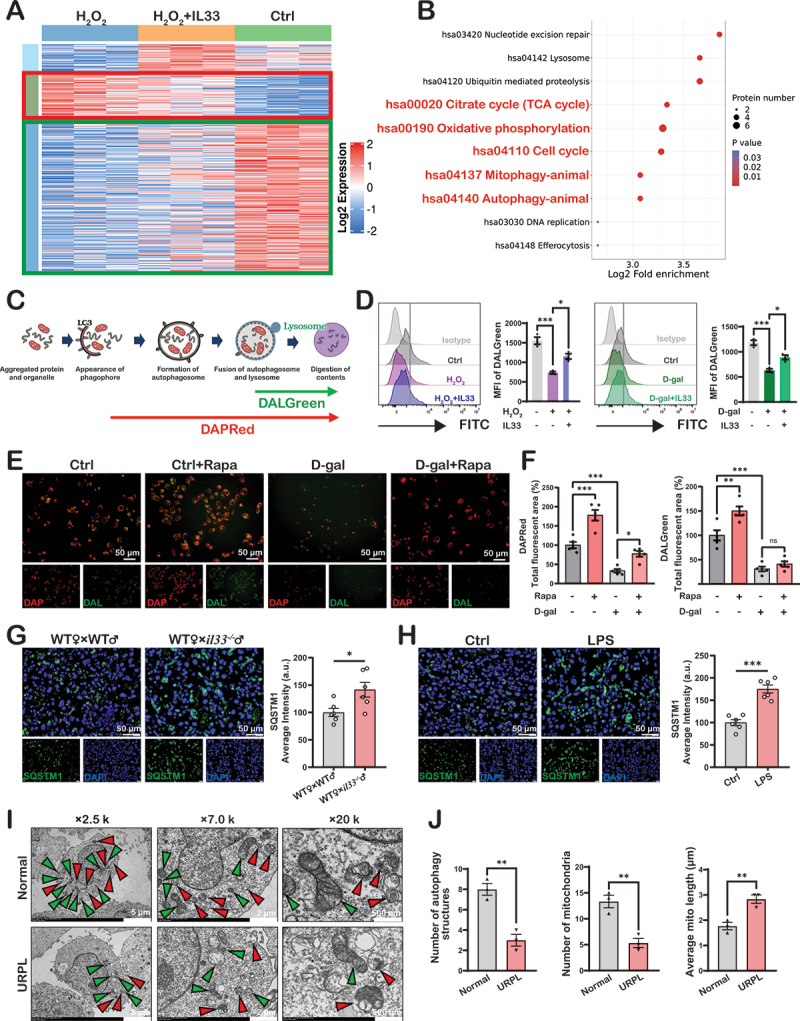
Trophoblast autophagy is impaired by IL33 deficiency-induced senescence. **(A**) Heatmap showed the relative expression changes of differential proteins in H_2_O_2_-induced cell senescence treated with or without IL33 (10 ng/mL). (**B**) KEGG enrichment analysis for the differentially expressed proteins identified in green box. (**C**) Schematic diagram of the different stages of autophagy detected by DAL/DAP fluorescence probes. (**D**) Autophagy flux was detected by the flow cytometry assay in HTR-8/SVneo cell senescence models treated with or without IL33 (10 ng/mL). (**E and F**) The level of autophagy was detected by the DAL/DAP fluorescence probes in HTR-8/SVneo cell senescence models treated with or without rapamycin (2 μM for 24 h). Scale bar: 50 μm. (**G**) The expression of SQSTM1 was detected by immunofluorescence staining in wt and *il33*^−/−^ mice (*n* = 6 per group). Scale bar: 50 μm. (**H**) The expression of SQSTM1 was detected by immunofluorescence staining in control and LPS-treated mice (*n* = 6 per group). Scale bar: 50 μm. (**I**) The number of autophagosomes and mitochondria in primary trophoblast cells from normal and URPL patients (*n* = 3 per group) were detected by transmission electron microscopy. Red arrows: autophagosomes, green arrows: mitochondria. (**J**) The number of autophagosomes, mitochondria and the average mitochondria length. Immunofluorescence staining results was quantified with average intensity. All data were generated using Image J. Statistical data were presented as mean ± SEM. *p < 0.05, **p < 0.01, ***p < 0.001, ns, no significant difference, using two-tailed Student’s t-test (G, H, j) and one-way ANOVA test (d, F). [Insert a space before “(a.U.).” If the red spheres in C are meant to be LC3 they should be on the membrane and on both sides of the phagophore.].

To further elucidate the mechanism by which IL33 mitigates trophoblast senescence, we performed a heatmap analysis of the differentially enriched proteins (Figure 3(A)). KEGG pathway enrichment analysis revealed that senescence was associated with significant downregulation of the TCA cycle, oxidative phosphorylation, macroautophagy/autophagy and cell cycle pathways, while IL33 treatment helped restore these processes (Figure 3(B)). Considering that autophagy plays an essential role in the process of pregnancy and is closely linked to cellular aging [18], we then utilized DAL/DAP probes to measure autophagic flux (Figure 3(C)). It turned out that autophagy was markedly suppressed in senescent trophoblasts, while IL33 treatment significantly rescued this defect (Figure 3(D)). Moreover, under normal conditions, the administration of autophagy inducer rapamycin significantly increased the number of autophagosomes and autolysosome. However, senescent trophoblast cells displayed a low level of basal autophagy. While rapamycin stimulated the formation of autophagosomes in these cells, the process failed to progress, indicating a disruption in autolysosome formation (Figure 3(E,F)).

Compared to controls, placenta from both *il33*^−/−^ mice and LPS-treated mice exhibited a marked accumulation of the autophagy substrate SQSTM1, suggesting a significant impairment of autophagic flux (Figure 3(G,H)). Subsequently, we collected villi to extract primary cells and observed them under electron microscopy. As shown, we observed obvious circular structures in normal villi, which are myeloid residues formed during the later stages of autophagy. The mitochondrial morphology in them was intact, with clear internal cristae visible. However, the number of autophagosomes and mitochondria was significantly reduced in URPL villi, with mitochondria appeared swollen (Figure 3(I,J)). These results suggest that both autophagic flux and mitochondrial performance are compromised in villi from patients with URPL.

### Senescent trophoblasts exhibit glycolytic activation and lactate accumulation, leading to impaired autophagy

**Figure 4.**
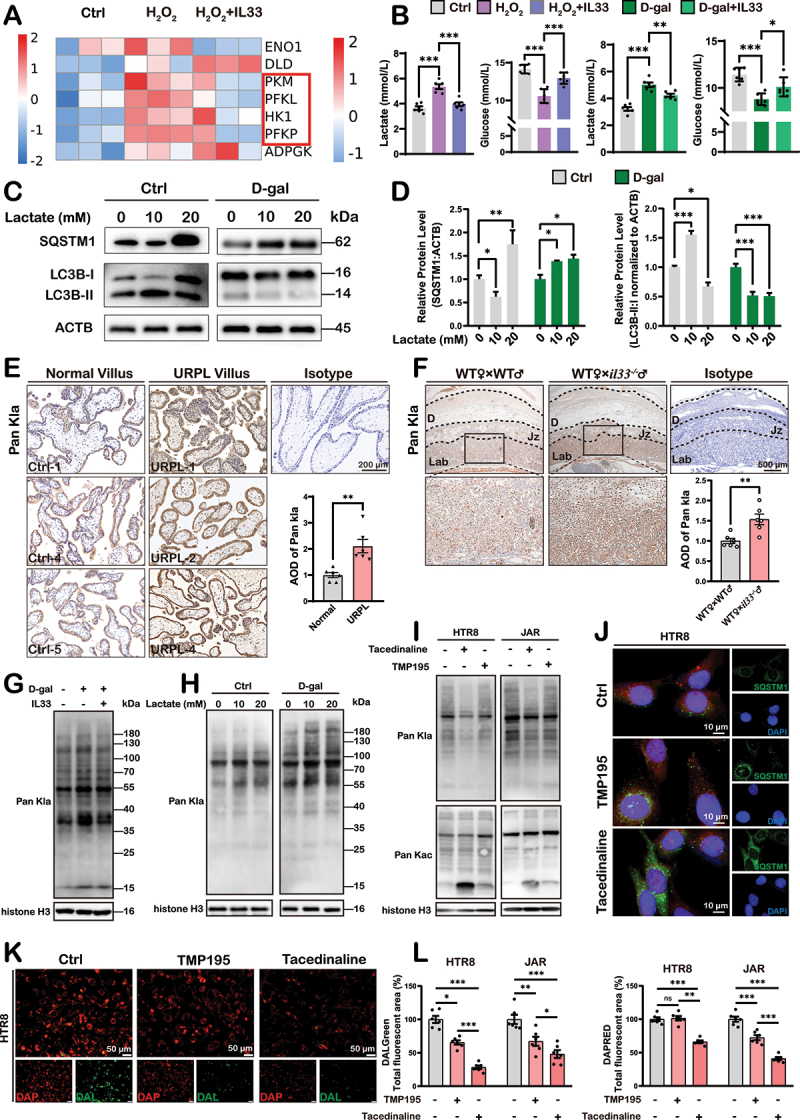
Senescence-induced glycolysis and lactate accumulation impair autophagy in trophoblasts. (**A**) Heatmap showed the relative expression changes of seven proteins in the glycolysis/gluconeogenesis pathway. (**B**) Glucose and lactate concentrations in the supernatant of senescent HTR-8/SVneo cells treated with or without IL33 (10 ng/mL). (**C and D**) Western blot for SQSTM1 or LC3B-II:I in normal and senescent HTR-8/SVneo cells treated with different concentration of lactate (0, 10, 20 mM for 48 h). Relative expression levels of proteins were standardized using internal reference ACTB. (**E**) The expression of lactylation level was analyzed by immunohistochemistry staining in normal and URPL villi (*n* = 6 per group). Scale bar: 200 μm. (**F**) The expression of lactylation level was analyzed by immunohistochemistry staining in wt and *il33*^−/−^ mice (*n* = 6 per group). Scale bar: 500 μm. (**G**) Western blot for lactylation level in HTR-8/SVneo cell senescence models treated with or without IL33 (10 ng/mL). Relative expression levels of proteins were standardized using internal reference histone H3. (**H**) Western blot for lactylation level in normal and senescent HTR-8/SVneo cells treated with different concentration of lactate (0, 10, 20 mM for 48 h). Relative expression levels of proteins were standardized using internal reference histone H3. (**I**) Western blot for lactylation and acetylation level in trophoblast cells treated with tacedinaline (5 μM, 48 h) or TMP195 (5 μM, 48 h). Relative expression levels of proteins were standardized using internal reference histone H3. (**J**) The expression of SQSTM1 was detected by immunofluorescence in HTR-8/SVneo cells treated with tacedinaline (5 μM, 48 h) or TMP195 (5 μM, 48 h). Scale bar: 10 μm. (**K and L**) The level of autophagy was detected by the DAL/DAP fluorescence probes in HTR-8/SVneo cells treated with tacedinaline (5 μM, 48 h) or TMP195 (5 μM, 48 h). Scale bar: 50 μm. Immunofluorescence staining results was quantified with average intensity. All data were generated using ImageJ. Statistical data were presented as mean ± SEM. *p < 0.05, **p < 0.01, ***p < 0.001, ns, no significant difference, using two-tailed Student’s t-test (e, F) and one-way ANOVA test (b, d, L).

Following H_2_O_2_-induced senescence, the expression levels of seven proteins involved in glycolysis/gluconeogenesis pathway were upregulated, and subsequently downregulated after IL33 supplementation. These included three key glycolytic enzymes: hexokinase, phosphofructokinase and pyruvate kinase (Figure 4(A)). Moreover, senescent cells showed decreased glucose levels and increased lactate levels in the supernatant (Figure 4(B)). These results suggest that senescence leads to abnormal activation of glycolysis. To explore the impact of accumulated lactate on autophagy, we treated trophoblast cells with varying concentrations of lactate. To exclude the confounding effect of lactate acidity, the pH of each lactate-containing solution was adjusted to approximately 7.4 using NaOH. In normal trophoblast cells, 10 mM lactate appeared to enhance autophagic activity, while higher concentrations led to its suppression, indicating a dose-dependent, biphasic regulatory role of lactate on autophagy. In contrast, accumulated lactate consistently exacerbated autophagic dysfunction in senescent cells (Figure 4(C,D)). To counteract this effect, we applied 2-deoxy-D-glucose (2-DG), a glycolysis inhibitor that reduces endogenous lactate production. Treatment with increasing concentrations of 2-DG partially restored autophagy levels in senescent trophoblast cells, while inhibition of glycolysis by 2-DG in normal trophoblast cells led to a reduction in autophagic activity (Fig. S4A, B), suggesting that a certain level of glycolytic metabolism and the resulting lactate is required to maintain basal autophagy.

We examined the lactylation levels in URPL villi, the placenta of *il33*^−/−^ mice, the placenta of LPS-treated mice and senescent trophoblast cells. The results showed a significant increase in lactylation levels compared to their respective control groups (Figure 4(E–G) and S4C). Exogenous lactate treatment elevated lactylation levels in both normal and senescent trophoblast cells, while administration of 2-DG suppressed lactate production and reduced lactylation levels (Figure 4(H) and S4D), confirming that lactate is a key regulator of protein lactylation. Histone Deacetylases (HDACs) are a class of enzymes responsible for removing acetyl groups from histones. Histone acetylation and lactylation can compete for the same lysine residues, indicating a potential regulatory interplay between these two post-translational modifications [19]. Therefore, we treated trophoblast cells with two types of histone deacetylase (HDAC) inhibitors to assess their effects on lactylation. It turned out that class I HDAC inhibitor (tacedinaline) significantly inhibited lactylation while promoting acetylation, whereas class II HDAC inhibitor (TMP195) only enhanced acetylation without affecting lactylation levels (Figure 4(I)). TMP195 treatment mildly impaired autophagy, suggesting that acetylation alone had limited effects. In contrast, tacedinaline treatment led to a marked suppression of autophagy, accompanied by a notable accumulation of SQSTM1 (Figure 4(J–L) and S4E-G). These findings support the notion that lactate regulates autophagy through lactylation, and that this modification plays a crucial role in maintaining an adequate level of autophagy in trophoblast cells.

### SNAP29 undergoes lactylation at lysine 169, modulating trophoblast autophagy and invasion

**Figure 5.**
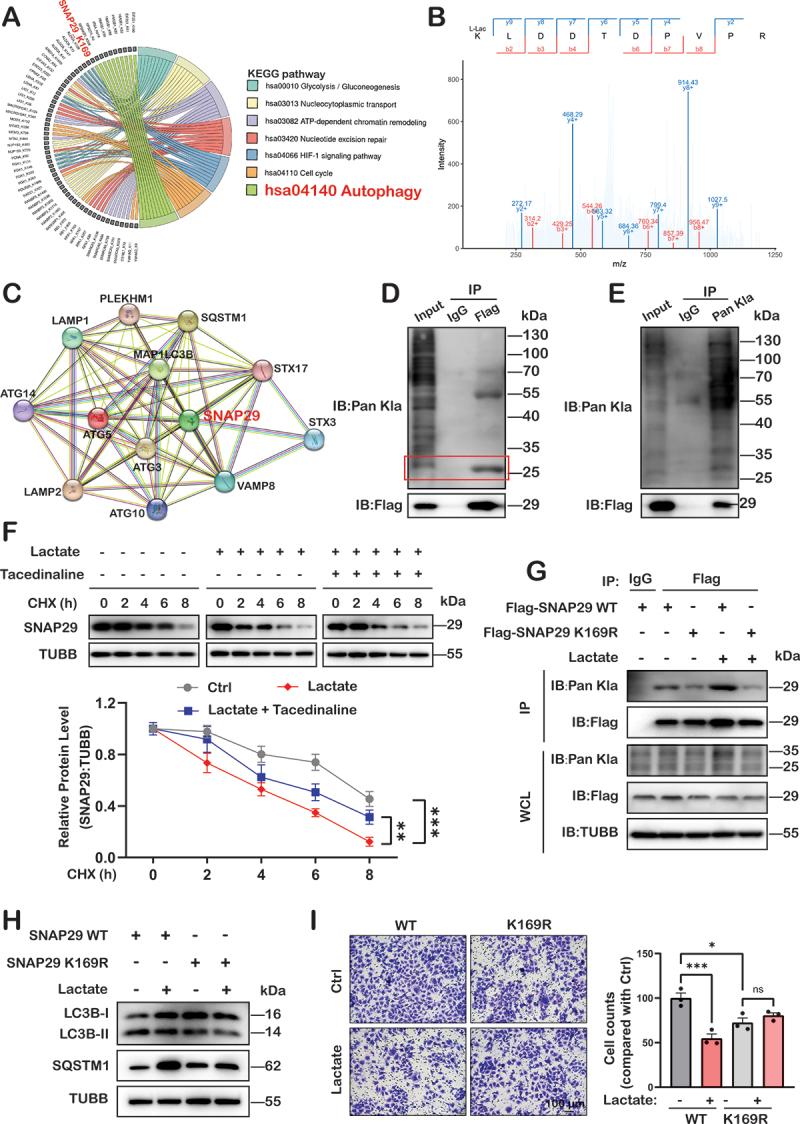
Senescence impairs trophoblast autophagy via enhanced lactylation of SNAP29 at K169. (**A**) KEGG enrichment analysis of L-lactylated proteins identified in HTR-8/SVneo cells. (**B**) Representative MS/MS spectrum of the identified lactylated peptide. (**C**) Differentially expressed gene-encoded protein-protein interaction network based on the string database. (**D and E**) Co-immunoprecipitation was performed using Flag or pan lactylation antibodies in HEK-293T cells. (**F**) Western blot for SNAP29 in HTR-8/SVneo cells treated with CHX (30 μg/mL, 0–8 h). Relative expression levels of proteins were standardized using internal reference TUBB. (**G**) HEK-293T cells transfected with wt or K169R SNAP29 plasmids were treated with 20 mM lactate for 48 h, followed by co-immunoprecipitation using anti-Flag or pan-lactylation antibodies. (**H**) Western blot for SQSTM1 and LC3B-II:I in wt vs. K169R HTR-8/SVneo cells after lactate treatment (20 mM, 48 h). Relative expression levels of proteins were standardized using internal reference TUBB. (**I**) Matrigel invasion assay was performed in wt vs. K169R HTR-8/SVneo cells after lactate treatment (20 mM, 48 h). Scale bar: 100 μm. All data were generated using ImageJ. Statistical data were presented as mean ± SEM. *p < 0.05, **p < 0.01, ***p < 0.001, ns, no significant difference, using one-way ANOVA test.

We performed lactylation-focused omics sequencing on HTR-8/SVneo cells, and identified a total of 1390 lactylated proteins and 3498 modification sites (Fig. S5A). To comprehensively characterize their functional relevance, we subsequently conducted subcellular localization analysis and functional annotation of these identified proteins (Fig. S5B, C). KEGG pathway enrichment analysis revealed that the identified proteins were widely enriched in autophagy pathways. Among them, lysine 169 of the autophagy-related protein SNAP29 (synaptosome associated protein 29) was identified as a lactylation site with clear b/y ion coverage (Figure 5(A,B)). SNAP29 is known to play a crucial role in autophagy, particularly in regulating autophagosome-lysosome fusion [20]. PPI analysis revealed that SNAP29 is closely associated with multiple autophagy-related proteins, such as ATG5, ATG3, MAP1LC3B, SQSTM1 and VAMP8 (Figure 5(C)).

To further validate the lactylation of SNAP29, we transfected HEK-293T cells with a plasmid encoding Flag-tagged SNAP29. Co-immunoprecipitation (co-IP) using an anti-Flag antibody successfully enriched lactylated proteins (Figure 5(D)). To further validate this interaction, a reverse co-IP was performed using a pan-lactylation antibody to pull down lactylated proteins and Flag-SNAP29 was also detected in the immunoprecipitates (Figure 5(E)). These bidirectional Co-IP results consistently demonstrate that SNAP29 is a protein susceptible to lactylation modification. Building on these findings, we next examined whether lactylation extends to other autophagy-related proteins. Pan-lactylation immunoprecipitation followed by western blotting for ATG5, STX17 and VAMP8 revealed minimal or undetectable lactylation signals (Fig. S5D). These results indicate that, in our system, lactylation preferentially modifies SNAP29, rather than broadly targeting the autophagosome – lysosome fusion machinery.

To further analyze whether lactylation influences the expression level of SNAP29, we assessed SNAP29 protein stability using cycloheximide (CHX), which blocks the synthesis of newly formed proteins in cells. In control group, SNAP29 remained relatively stable up to 6 h post-treatment, with a gradual decrease observed after 8 h. In contrast, treatment with lactate (pH = 7.4 adjusted with NaOH) resulted in a marked reduction in SNAP29 protein levels as early as 2 h, with near-complete degradation by 8 h, suggesting that elevated lactylation level promotes protein degradation. However, co-treatment with tacedinaline stabilized SNAP29 at 2 h and delayed its degradation between 4–8 h. At the 8 h time point, SNAP29 levels were lowest in the lactate-treated group, while the presence of the tacedinaline partially rescued its stability (Figure 5(F)). These observations suggest that enhanced lactylation promotes SNAP29 destabilization, however, the degradation pathway responsible for this effect remained to be elucidated. To clarify the mechanism of SNAP29 degradation, we performed CHX chase assays in the presence of either the proteasome inhibitor MG132 or the lysosomal inhibitor bafilomycin A_1_ (BafA1). As shown in Figure S5E, MG132 markedly attenuated CHX-induced SNAP29 degradation, whereas BafA1 treatment did not confer a detectable protective effect. These results indicate that SNAP29 is predominantly degraded via a proteasome-dependent pathway, rather than through lysosomal degradation.

In addition, to determine whether lysine 169 (K169) serves as the primary site of SNAP29 lactylation, we constructed a K169R mutant by replacing lysine with arginine, thereby mimicking a de-lactylated state. Co-immunoprecipitation assays revealed that the lactylation level of the K169R mutant was markedly reduced compared to the WT SNAP29. Furthermore, upon exogenous lactate treatment, lactylation levels were significantly elevated in the WT group but remained unchanged in the K169R mutant (Figure 5(G)). Treatment with 20 mM lactate significantly inhibited autophagy in WT HTR-8/SVneo cells, however, in K169R mutant cells with already diminished basal autophagy, additional lactate exposure had no further inhibitory effect (Figure 5(H) and S5F-G). Moreover, lactate treatment markedly impaired the invasive capacity of WT cells. Interestingly, the K169R mutation alone was sufficient to reduce cell invasiveness, and this effect was not further exacerbated by lactate exposure (Figure 5(I)). Together, these findings collectively highlight lysine 169 as a critical functional site for SNAP29 lactylation.

### Increased SNAP29 lactylation impairs autophagy and invasive capacity of trophoblasts and contribute to URPL

**Figure 6.**
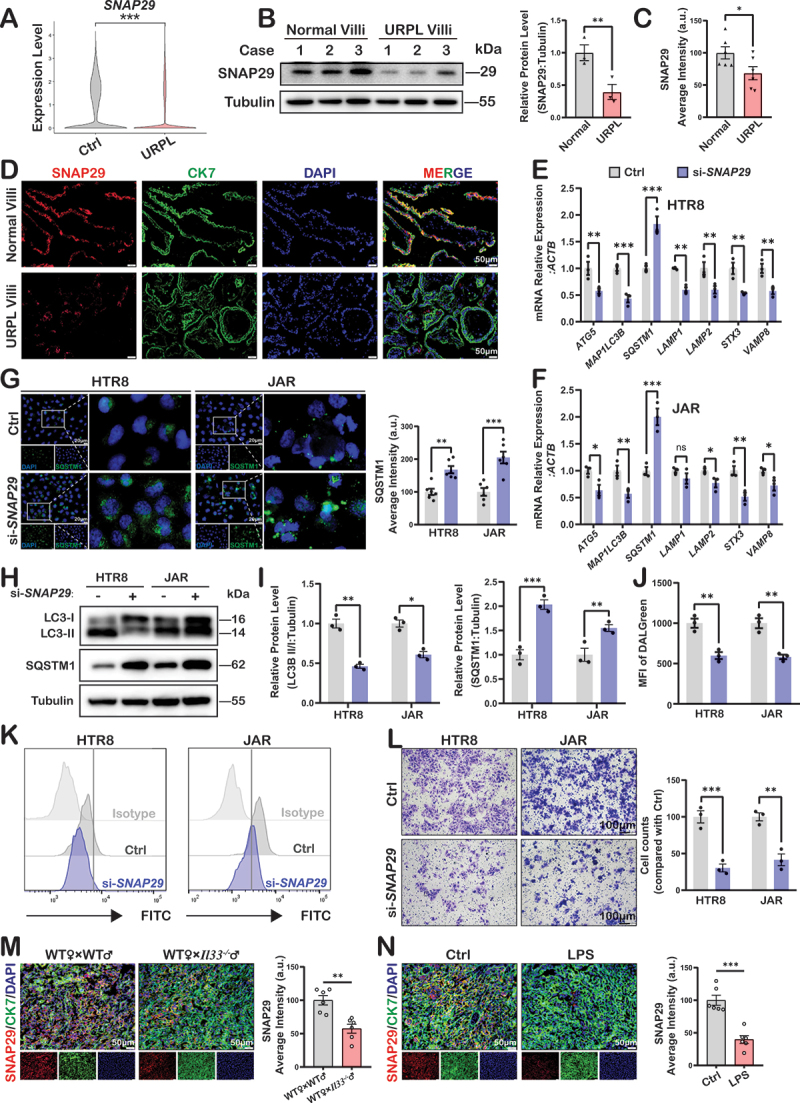
Lactylation of SNAP29 suppresses autophagy and compromises trophoblast invasive capacity. (A) Violin plot showing the expression of *SNAP29* in GSE214607 dataset. (B) Western blot for SNAP29 in normal and URPL villous tissues (*n* = 3 per group). Relative expression levels of proteins were standardized using internal reference TUBB. (C and D) The expression of SNAP29 was analyzed by immunofluorescence staining in normal and URPL villous tissues (*n* = 6 per group). Scale bar: 50 μm. (E and F) Relative mRNA expression levels of autophagy-related genes in HTR-8/SVneo and jar cells transfected with control or si-*SNAP29*. (G) The expression of SQSTM1 was analyzed by immunofluorescence staining in HTR-8/SVneo and jar cells transfected with control or si-*SNAP29*. Scale bar: 20 μm. (H and I) Western blot for SQSTM1 and LC3B-II:I in HTR-8/SVneo and jar cells transfected with control or si-*SNAP29*. Relative expression levels of proteins were standardized using internal reference TUBB. (J and K) autophagy flux was detected by the flow cytometry assay in HTR-8/SVneo and jar cells transfected with control or si-*SNAP29*. (L) Matrigel invasion assay was performed in HTR-8/SVneo and jar cells transfected with control or si-*SNAP29*. Scale bar: 100 μm. (M) The expression of SNAP29 was evaluated by immunofluorescence in wt and *il33*^−/−^ mice (*n* = 6 per group). Scale bar: 50 μm. (N) The expression of SNAP29 was evaluated by immunofluorescence in control and LPS-treated mice (*n* = 6 per group). Scale bar: 50 μm. The immunofluorescence staining was quantified with average intensity. All data were generated using ImageJ. Statistical data were presented as mean ± SEM. *p < 0.05, **p < 0.01, ***p < 0.001, using two-tailed Student’s t-test. [use italics for mRNAs and for siRnas.].

Based on publicly available single-cell RNA-sequencing data, SNAP29 expression was markedly reduced in villous tissues from URPL patients (Figure 6(A)). To validate this finding at the protein level, we collected villous tissues from both healthy pregnancies and URPL patients. Western blot analysis confirmed a significant downregulation of SNAP29 protein in URPL samples compared to controls (Figure 6(B)). In addition, immunofluorescence staining revealed that SNAP29 was broadly localized to the cytoplasm and plasma membrane of trophoblast cells, partially colocalizing with the trophoblast marker KRT7/CK7 (Figure 6(C,D)).

To investigate whether SNAP29 plays a regulatory role in trophoblast autophagy, we constructed negative control and SNAP29-silenced (si-*SNAP29*) plasmids. Knockdown of SNAP29 led to a marked downregulation of several autophagy-related genes, including MAP1LC3B, SQSTM1 and LAMP1 (Figure 6(E,F)). Western blot analysis further revealed a significant accumulation of the autophagy substrate SQSTM1 (Figure 6(G–I)), indicating impaired autophagic degradation. Consistently, DALGreen fluorescence was substantially reduced in si-*SNAP29* cells (Figure 6(J,K)), suggesting a defect in autolysosome formation.

In addition, SNAP29 knockdown significantly reduced the invasive capacity of both HTR-8/SVneo and JAR cells (Figure 6(L)). This phenotype is consistent with our previous observations in cells transfected with SNAP29^K169R^ mutant. To further explore the physiological relevance of SNAP29 in vivo, we examined its expression in placental tissues. Compared with controls, placentas from both *il33*^*− /−*^ and LPS-treated mice showed significantly reduced SNAP29 levels (Figure 6(M,N)). Taken together, these results highlight the essential role of SNAP29 in maintaining autophagic homeostasis and trophoblast invasiveness during placental development, and underscore the importance of its lactylation-mediated stabilization under physiological and pathological conditions.

### Anti-senescence interventions alleviate placental dysfunction and improve pregnancy outcomes in mice

To investigate whether targeting cellular senescence could improve pregnancy outcomes, we assessed the therapeutic potential of two different anti-aging strategies in mice miscarriage models. Metformin, a widely studied anti-aging agent, is known for its roles in modulating mitochondrial function and metabolic homeostasis [21]. The combination of Dasatinib and Quercetin (D+Q), a classical senolytic cocktail, selectively eliminates senescent cells [22]. These interventions were applied in two distinct senescence-related models of pregnancy loss: the *il33* knockout model, which exhibits premature placental aging due to the loss of IL33, a key immune regulatory factor; and the LPS-induced inflammation model, which mimics inflammation-driven senescence within the placental microenvironment.

**Figure 7.**
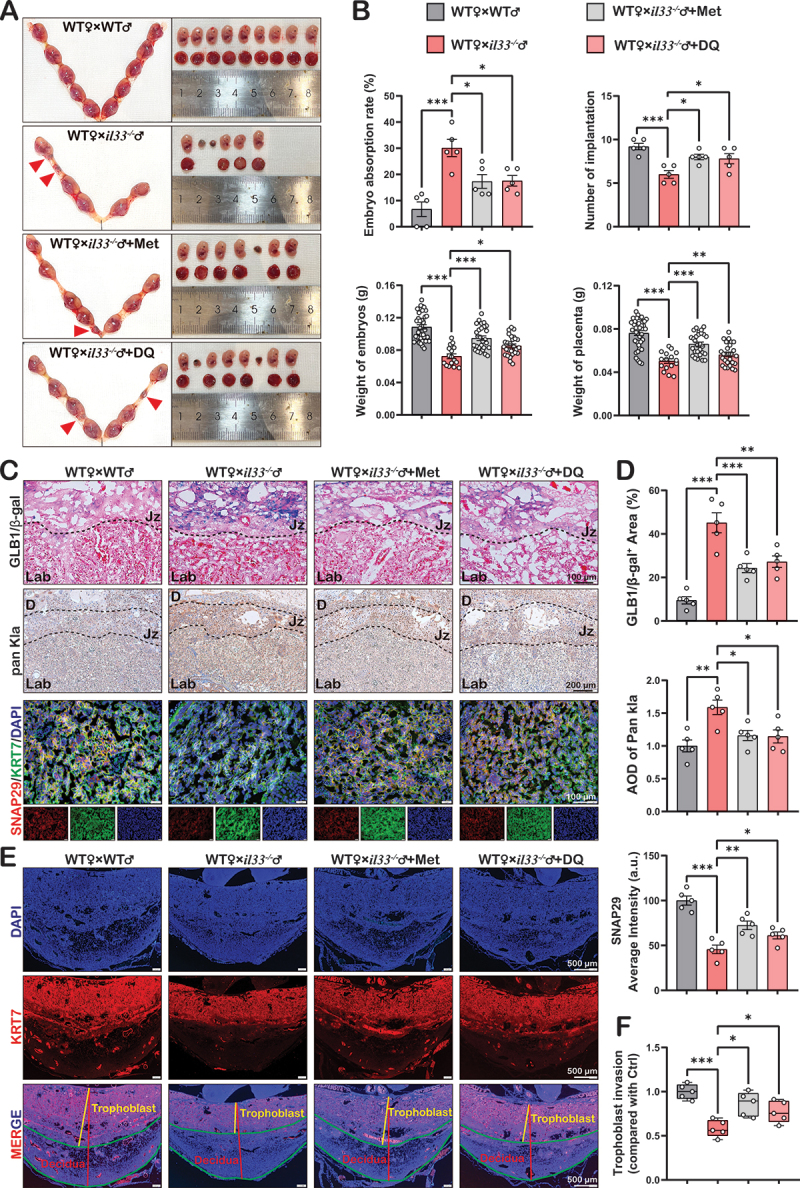
Anti-senescence interventions alleviate placental dysfunction and rescue the pregnancy loss in IL33-deficient mice. (**A and B**) The pregnancy outcomes at gestational day 13.5 were assessed in the control (*n* = 5), *il33*^−/−^ (*n* = 5), *il33*^−/−^ + metformin (*n* = 5), and *il33*^−/−^ + DQ (*n* = 5) groups. Embryo resorption rate, number of blastocyst implantation, weight of placenta and embryo were observed in mice. (**C and D**) Placental senescence was assessed by GLB1/β-galactosidase staining, lactylation levels were examined by immunohistochemistry, and SNAP29 expression was evaluated by immunofluorescence (*n* = 5 per group). The immunohistochemistry staining was quantified with average optical density (AOD) and immunofluorescence staining was quantified with average intensity. Scale bar: 100 μm and 200 μm. (**E and F**) The depth of trophoblast infiltration in the placenta was assessed by immunofluorescence (*n* = 5 per group). The ratio of trophoblast infiltration (yellow line) to the depth of the entire uterus (red line) was analyzed. Scale bar: 500 μm. All data were generated using ImageJ. Statistical data were presented as mean ± SEM. *p < 0.05, **p < 0.01, ***p < 0.001, using one-way ANOVA test.

As expected, both metformin and D+Q treatment significantly reduced embryo resorption rates, increased embryo and placental weights, and improved overall pregnancy outcomes in IL33-deficient and LPS-induced inflammation models (Figure 7(A,B) and S6A-B). In addition, both interventions effectively attenuated placental senescence and lactylation levels, and restored the expression of SNAP29 (Figure 7(C,D) and S6C-D). Notably, metformin and D+Q treatment also enhanced the depth of trophoblast infiltration into the maternal decidua, indicating improved placental invasiveness (Figure 7(E,F) and S6E-F). Given previous reports that metformin exposure can affect placental mitochondrial function [23], we further examined whether metformin modulates mitochondrial homeostasis in senescent placentas. In both *il33*^−/−^ and LPS-treated placentas, qPCR analysis revealed reduced expression of *Ppargc1a*, *Opa1*, and *Mfn2*, along with increased levels of *Dnm1l* and *Mff*—indicative of impaired mitochondrial biogenesis and a shift toward fission. Immunofluorescence staining further showed decreased expression of mitochondrial markers TOMM20 and TFAM. Notably, these alterations were largely reversed following metformin treatment, suggesting that metformin can alleviate senescence-associated mitochondrial dysfunction in the placenta (Fig. S7A-F). Consistent findings across both *il33* knockout and LPS-treated groups confirm the generalizable benefits of these anti-aging strategies. Taken together, these findings indicate that metformin and D+Q alleviate placental dysfunction by suppressing trophoblast senescence, thereby reducing the risk of pregnancy loss in senescence-related models.

## Discussion

Premature cellular senescence is increasingly viewed as a unifying mechanism behind many obstetric pathologies, such as preeclampsia and fetal growth restriction [24,25]. As a nuclear alarmin-like cytokine of the IL1 family, IL33 has been increasingly recognized for its regulatory roles beyond immune modulation [26]. Here, we found that IL33 deficiency in vivo leads to excessive trophoblast senescence and impaired placental development. These findings suggest that although IL33 is primarily derived from maternal tissues, its regulatory effects extend to the fetal-derived trophoblasts. As known, IL33 has been reported to enhance macrophage polarization and facilitate tissue repair following skin injury induced by radiation-associated senescent fibroblasts [27]. In renal epithelial cells, IL33 was shown to modulate cell cycle regulators and improve tubular senescence and injury [28]. Furthermore, IL33 induces chemotaxis of VCAM1^+^ microglia, which subsequently interact with ApoE, thereby facilitating β-amyloid clearance and alleviating the progression of Alzheimer disease [29]. These findings collectively suggest that IL33 serves as a crucial regulator of cellular senescence, exerting its protective roles via anti-inflammatory signaling, metabolic reprogramming, and immune modulation. However, the precise downstream effectors of IL33 and its role in post-translational modifications remain largely unknown. Our study reveals, for the first time, that IL33 May exert its anti-senescent effect by modulating SNAP29 lactylation and autophagy in trophoblasts – thereby linking immune signaling to metabolic control of placental aging.

Autophagy plays a pivotal role in maintaining trophoblast homeostasis throughout pregnancy, and insufficient autophagy has been identified as a key contributor to RPL [30,31]. Notably, impaired autophagy is also a hallmark of cellular senescence, and reactivation of autophagic pathways is increasingly recognized as a promising therapeutic strategy in the fields of anti-aging and regenerative medicine [18,32]. Our data revealed that senescent trophoblasts display enhanced glycolytic activity and lactate accumulation, which further impairs autophagic flux and exacerbates autophagy dysfunction. This aligns with previous studies showing that senescent cells undergo extensive metabolic remodeling [33,34]. It has been reported that senescent cardiomyocytes exhibit reduced oxidative phosphorylation and a compensatory increase in glycolysis, resulting in energy deficits and age-related cardiac dysfunction [35]. Naoya et.al reported that hyperactive glycolysis is a metabolic signature of aged and diabetic β cells, which may be mediated by the increased expression of nicotinamide mononucleotide adenylyl transferase 2 [36]. Although the maternal – fetal interface is relatively hypoxic and glycolysis is naturally upregulated during implantation [37], accumulation of excessive lactate undoubtedly have detrimental effects on placental autophagy and development. Given the essential role of autophagy in trophoblast survival, differentiation, and invasion during early pregnancy [38], these findings collectively underscore the central role of the IL33–senescence – autophagy axis in safeguarding placental function. Therefore, therapeutic strategies aimed at restoring autophagy may offer novel avenues for alleviating placental insufficiency, especially in disorders characterized by premature trophoblast senescence.

Lactylation (Kla), first characterized in 2019, is a novel post-translational modification derived from glycolysis by-products [39]. In this study, we identified lysine 169 (K169) of SNAP29 (synaptosome associated protein 29) as a previously unreported lactylation site. SNAP29 is a key member of the SNARE family and plays a central role in mediating autophagosome – lysosome fusion. This fusion event relies on the coordinated interaction between Q-SNAREs localized on the autophagosome membrane, such as STX17 (syntaxin 17), and R-SNAREs situated on the lysosomal membrane, including VAMP7 (vesicle associated membrane protein 7) and VAMP8. Acting as a molecular “zipper,” SNAP29 bridges these SNARE components, bringing the opposing membranes into close apposition to enable their fusion [20,40]. Our study not only broadens our understanding of post-translational control over SNAP29, but also uncovers a previously unrecognized metabolic – autophagy axis in which aberrant lactylation precipitates placental dysfunction. Several additional lactylated proteins detected in our dataset have been independently linked to autophagy and trophoblast biology, implying that their lactylation could indirectly reinforce the observed phenotype. Consequently, whether other autophagy-related proteins are subject to lactylation – and, if so, how such modifications modulate the autophagic flux – remains a critical, unresolved question that merits systematic investigation. For instance, PGK1 (phosphoglycerate kinase 1) has been reported to suppress autophagy-mediated cell death and promote liver cancer cells proliferation via phosphorylation of AKT1S1/PRAS40, a key downstream target of the MTORC1 pathway [41]. Given the central role of MTOR signaling in autophagy regulation, it is plausible that Kla modification may also alter PGK1 activity or protein – protein interactions, thereby indirectly modulating autophagic flux.

Metformin, known to modulate mitochondrial function and AMPK signaling, has been shown to delay senescence and promote autophagy in various contexts [42,43]. Dasatinib and quercetin (D+Q) selectively eliminate senescent cells by targeting anti-apoptotic pathways and are considered classical senolytics [44,45]. Our results demonstrate that both interventions significantly alleviate placental dysfunction and improve pregnancy outcomes in mice. Interestingly, metformin demonstrated slightly more robust effects in preventing pregnancy loss and improving placental function. This may be attributed to the fact that we adopted a well-established dosing regimen widely used in pregnancy-related studies, ensuring both efficacy and safety [13,46]. In contrast, while D+Q has shown potent senolytic activity in various disease models, most published protocols employ higher doses optimized for maximally clearing senescent cells in non-pregnant settings [47,48]. In our study, we carefully selected a moderate and pregnancy-safe dose of D+Q, with the goal of alleviating senescence-associated pathology without disrupting physiological processes essential for successful gestation. Collectively, these findings highlight the therapeutic potential of anti-senescence interventions in mitigating placental dysfunction and pregnancy loss.

**Figure 8.**
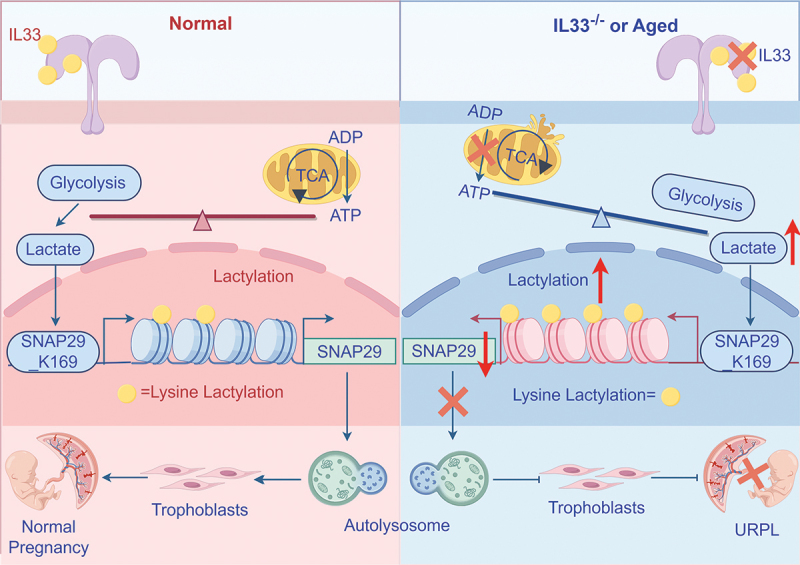
The mechanism of IL33 deficiency induced trophoblast excessive senescence in recurrent pregnancy loss. Under physiological conditions, IL33 acts as an upstream regulator to maintain trophoblast senescence homeostasis, supporting normal placental development. In pathological states such as IL33 deficiency or inflammation, trophoblasts undergo excessive senescence, accompanied by hyperactive glycolysis, lactate accumulation, and mitochondrial dysfunction. Elevated lactate induces K169 lactylation of SNAP29, leading to impaired autophagic flux and downregulated trophoblast function. These changes ultimately disrupt placental development and increase the risk of URPL. This figure was drawn by Figdraw (www.figdraw.com). IL33: interleukin 33; SNAP29: synaptosome associated protein 29; URPL: unexplained recurrent pregnancy loss.

In summary, as shown in Figure 8, we propose that IL33 serves as an upstream regulator that maintains trophoblast homeostasis during normal pregnancy. However, under pathological conditions such as IL33 deficiency or inflammatory stimuli, trophoblasts may undergo excessive senescence. This process is accompanied by disrupted metabolic balance, characterized by hyperactivated glycolysis and lactate accumulation. Elevated lactate levels trigger lysine 169 (K169) lactylation of the autophagy-related protein SNAP29, resulting in its destabilization and downregulation. As a result, autophagic flux is impaired, further compromising trophoblasts invasiveness and implantation capability. These cellular dysfunctions ultimately hinder placental development and increase the risk of adverse pregnancy outcomes. Together, our findings not only elucidate a previously unrecognized IL33-SNAP29-lactylation axis as a key driver of placental aging, but also reveal the potential of targeting IL33–related pathways to preserve placental homeostasis and improve pregnancy outcomes under pathological conditions. By bridging mechanistic insights with in vivo validation, this work advances the framework for precision anti-senescence therapy in reproductive medicine, with potential implications extending to other aging-associated disorders.

## Materials and methods

### Patients and sample collection

This study was approved by the Human Research Ethics Committee of the Obstetrics and Gynecology Hospital of Fudan University (No. kyy2023-125), and all the participants provided written informed consent. Villi and decidua were obtained from healthy pregnant females (*n* = 25) who had undergone selective termination in the first trimester of pregnancy (gestational age, 5–10 weeks; maternal age, 25–35 years) or from patients (*n* = 12) with URPL (experiencing two or more consecutive spontaneous miscarriages before the 24th week of pregnancy) caused by non-endocrine or non-genetic factors (gestational age, 7–9 weeks; maternal age, 25–35 years). All cases were histologically verified based on established criteria. Pregnancies were confirmed using ultrasonography and blood tests. Females who had experienced spontaneous miscarriages attributed to endocrine, anatomical, or genetic issues, or due to any infection, were excluded from the study. Samples were collected under sterile conditions and transported to the laboratory within 30 min of surgery in DMEM/F12 (HyClone, SH30023.01) containing 10% fetal bovine serum (FBS; Gibco, 10,091–148).

### Animals

C57BL/6 mice (5–6 weeks old) were purchased from GemPharmatech Co., Ltd. (Shanghai, China). All mice were housed in SPF grade animal rooms. After 2 weeks of adaptive feeding, we performed further experiments. The Animal Care and use Committee of Fudan University approved all the animal protocols (No.2023-FCYY-62JZS).

C57BL/6 female mice were mated with C57BL/6 male mice (2:1). On gestational day 8.5, lipopolysaccharide (LPS, 0.1 mg/kg; Sigma-Aldrich, L2880) was administered via intraperitoneal injection to the experimental group, while the control group received an equivalent volume of phosphate-buffered saline (PBS; Servicebio, G4202).

The *il33* knockout (*il33*^−/−^) mice were constructed by Shanghai Model Organisms Center, Inc. (Shanghai, China). To exclude the influence of maternal decidua on the results, female wild-type mice were mated with male wild-type mice as the control group, female wild-type mice were mated with male *il33*^−/−^ mice as the experimental group.

From gestational day 0, pregnant mice in the experimental group were provided with drinking water containing either metformin (250 mg/kg/d; Sigma-Aldrich, PHR1084) or a combination of dasatinib (D, 2 mg/kg/d; MedChem Express, HY-10181) and quercetin (Q, 20 mg/kg/d; MedChem Express, HY-18085). To enhance the solubility of D+Q, 1% (w:v) sulfobutyl ether-β-cyclodextrin (SBE-β-CD; Aladdin, C125030) was used as a solubilizing agent. The solutions were protected from light throughout the treatment period. Additionally, 1% (w:v) sucrose (Aladdin, S112226) was added to all drinking water preparations to improve palatability. Drinking solutions were freshly prepared and replaced every two days to ensure stability and consistent dosing. On gestational day 13.5, the mice were sacrificed to evaluate pregnancy outcomes, including the number of implanted embryos, embryo resorption rate, fetal weight, and placental weight.

### Cell culture and treatment

HTR-8/SVneo and JAR cells were obtained from American Type Culture Collection (CRL-3271, HTB-144) and cultured in DMEM/F12 medium supplemented with 10% FBS, 1% penicillin-streptomycin (Procell, PB180121).

To induce senescence, HTR-8/SVneo cells were treated with hydrogen peroxide (H_2_O_2_, 50 μM, 4 h; Sigma-Aldrich, 323,381) and subsequently incubated in fresh complete culture medium for 4 days, or with D-galactose (D-gal, 200 mM, 4 days; Sigma-Aldrich, G5388). JAR cells were treated with etoposide (VP, 2 μM, 12 h; MedChem Express, HY-13629) and subsequently incubated in fresh complete culture medium for 4 days, or with D-galactose (D-gal, 200 mM, 6 days; Sigma-Aldrich, G5388).

IL33 (10 ng/mL; PeproTech, 200–33), rapamycin (2 μM, 24 h; Sigma-Aldrich, V900930), lactate (10 or 20 mM, 48 h; Sigma-Aldrich, L6661), tacedinaline (5 μM, 48 h; MedChem Express, HY-50934), TMP195 (5 μM, 48 h; MedChem Express, HY-18361), 2-deoxy-D-glucose (2-DG; 5 or 10 mM, 48 h; MedChem Expres, HY-13966s), Cycloheximide (CHX, 30 μg/mL, 0–8 h; MedChem Express, HY-12320), MG132 (10 µM, 9 h; MedChem Express, HY-13259), bafilomycin A_1_ (BafA1, 100 nM, 9 h; MedChem Express, HY-100558) were used.

### Immunohistochemistry

Paraffin sections of samples were baked at 60°C for 2 h, deparaffinized with dimethylbenzene and dehydrated in graded ethanol. After being boiled in Tris – EDTA buffer or sodium citrate for antigen retrieval, tissue sections were incubated with 3% H_2_O_2_ and 5% bovine serum albumin to block endogenous peroxidase activity. The samples were subsequently incubated with rabbit IL33 (1:1000; Abcam, human: ab207737; mouse: ab187060), rabbit ST2 (1:500; Abcam, ab194113), rabbit IL6 (1:200; Affinity, DF6087), rabbit CDKN1A/p21 (1:200; Abcam, human: ab109520; mouse: ab188224), rabbit Pan Kla (1:1000; PTM BIO, PTM-1401RM), or rabbit immunoglobulin G (IgG) isotype control (1:1000; Abcam, ab125938) overnight at 4°C in a humid chamber. After washing three times with PBS, the sections were overlaid with peroxidase-conjugated goat antirabbit IgG, the re-action was developed using 3, 3-diaminobenzidine (DAB; Gene Tech, GK500710), and the sections were counterstained with hematoxylin. The immunohistochemical staining was quantified with integrated optical density values generated using ImageJ (National Institute of Mental Health, USA).

### Immunofluorescence

Baking, dewaxing, rehydration, antigen retrieval, and serum blockade were performed as described above. The samples were subsequently incubated with rabbit KRT7/CK7 (keratin 7, 1:2000; Abcam, ab181598) or mouse KRT7 (1:1000; Abcam, ab216016) or rabbit SQSTM1/p62 (1:1000; Abcam, ab109012) or rabbit SNAP29 (1:200; Abcam, ab181151) or rabbit TOMM20 (1:200; Abcam, ab186735) or rabbit TFAM (1:100, Abcam, ab307302) overnight at 4°C in a humid chamber. After washing three times with PBS, the sections were incubated with secondary goat anti-rabbit (1:1000; Abcam, ab150080) or goat anti-mouse (1:1000; Abcam, ab150113) antibody for 1 h at room temperature. Nuclei were stained with 4',6-diamidino-2-phenylindole (DAPI; Beyotime, P0131).

### Senescence‑associated beta‑galactosidase (SA‑β‑gal) staining

SA-GLB1/β-gal staining was conducted using a Senescence β-Galactosidase Staining Kit (Beyotime, C0602) following the manufacturer’s instructions. Fresh samples were embedded in O.C.T compound (Servicebio, G6059), cryo-frozen, and sectioned into 5-μm slices with cryostat. Fresh tissues (villi and decidua), cryo-sections, or cells were fixed and incubated with SA-β-staining buffer at 37°C overnight in a CO_2_-free environment. Cryo-sections were subsequently washed twice with PBS and counterstained with eosin for 30 s. The stained tissues and cells were directly observed under an inverted microscope, with senescent tissues and cells were appearing as blue-green-stained structures.

### Cell invasion assays

After cell senescence was induced (with or without 10 ng/mL IL33 supplemented), the invasive ability of the HTR-8/SVneo and JAR cells were analyzed via a Matrigel (Corning, 356,234) invasion assay. Matrigel was diluted with DMEM/F12 at a ratio of 1:8, and 100 μL diluted Matrigel was added to the upper chamber. Put the plate into a 37°C incubator for 2 h, removed the excess liquid. 200 μL (HTR‑8/SVneo or JAR cells, 1 × 10^5^ cells/well) DMEM/F‑12 suspension without 10% FBS was added to the upper chamber, and 500 μL DMEM/F‑12 containing 10% FBS was added to the lower chamber. After 48 h, the upper chamber medium and non-penetrating cells were gently wiped off. Cells were fixed with 4%PFA for 30 min and stained with crystal violet for 20 min. Random photographs were taken under an inverted microscope (Leica, Munich, Germany), and 4 visual fields were counted in each chamber. The number of invaded cells was counted using ImageJ.

### RNA extraction and reverse transcription quantitative real-time polymerase chain reaction (RT-qPCR)

The total RNA was extracted using TRIzol® reagent (Invitrogen, 15,596,018). RNA extraction was carried out on HTR-8/SVneo, JAR cells and mouse placental tissues. The RNA was reverse-transcribed into cDNA using a Prime Script RT reagent kit (Takara, RR036A). The qPCR was performed using an SYBR Premix Ex Taq kit (Takara, RR820A) and analyzed using an ABI Prism 7900 Fast Sequence Detection system (Thermo Fisher Scientific, Inc.). The primer sequences of these genes are provided in Table S1 and S2. The fold change in the transcriptional expression of the above genes was calculated using the 2^−ΔΔCt^ method, and each sample was analyzed in three replicate wells. The relative mRNA expression levels were normalized to *ACTB/β-actin* for human samples and *Actb* for mouse samples.

### ANXA5/annexin V-PI apoptosis assay

After cell senescence was induced (with or without 10 ng/mL IL33 supplemented), cell apoptosis was assessed by flow cytometry with ANXA5-FITC Apoptosis Detection Kit (Beyotime, C1062). HTR-8/SVneo and JAR cells (5 × 10^5^ cells) were trypsinized using 0.25% trypsin without EDTA. Collected, washed and resuspended cells with 195 μL binding buffer, followed by incubation with 5 μL ANXA5-FITC and 10 μL PI at room temperature for 15 min in the dark. Samples were measured with CytoFLEX flow cytometer (Beckman Coulter, Inc., Brea, CA, USA). The data was analyzed with FlowJo (version 10.8.1, BD Life Sciences-Biosciences, Ashland, OR, USA). ANXA5 ^+^ PI^−^ cells were in the early stage of apoptosis and ANXA5^+^ PI^+^ cells were late apoptotic cells.

### DALGreen/Dapred autophagy detection

After cell senescence was induced (with or without 10 ng/mL IL33 supplemented), collected cells, reseeded on μ-slide 8 well (Ibidi, 80,827) and cultured overnight. The cells were washed with culture medium and then incubated at 37°C for 30 min with 200 μL working solution, containing 1 μM DALGreen and 0.2 μM DAPRed (Dojindo, Autophagic Flux Assay Kit A562). After washed with the culture medium twice, DALGreen/DAPRed was observed under fluorescence microscopy. Or the cells were washed with the culture medium twice, collected and resuspended in PBS. Samples were measured with CytoFLEX flow cytometer. Autophagy flux was analyzed with FlowJo.

### Measurement of the lactate and glucose concentration

The concentration of lactate was detected by a lactate assay kit (Dojindo, L256), and the concentration of glucose was detected by a glucose assay kit (Dojindo, G264). After cell senescence was induced (with or without 10 ng/mL IL33 supplemented), supernatants of HTR-8/SVneo cells were collected and detected according to the manufacturer’s instructions.

### The isolation and culture of the first-trimester human trophoblast cells

Primary human trophoblast cells were obtained from normal and URPL villous tissues. The villi were placed in a sterile petri dish, quickly rinsed and cleaned twice with PBS, and the membrane was carefully removed using ophthalmic scissors. The villi were then cut into approximately 1–2 mm^3^ pieces, digested with 0.25% trypsin – EDTA (NCM Biotech, C100C1) and 0.1% DNA enzyme (Sigma-Aldrich, DN25) at 37°C for 10 min. The samples were sieved through sterile gauze pads (pore diameter sizes: 100, 300, and 400 mesh), and the suspension was centrifuged at 250 × g for 8 min. The cells were then suspended in DMEM/F12, slowly added to a discontinuous gradient of 15%, 30%, 45% and 60% Percoll (GE Healthcare Life Sciences, 17–0891-01) bulk standard, and centrifuged at 450 × g for 20 min. The human trophoblast cells were at the 30–60% interface.

### Transmission electron microscopy detection

Normal and URPL primary human trophoblast cells were collected and fixed in 2.5% glutaraldehyde and post-fixed in 1% osmium tetroxide. The samples were dehydrated in an ascending series of alcohols, and then embedded in epoxy resin (Sigma-Aldrich, 45,345). The sections were cut, stained with uranyl acetate and lead citrate, and then examined under transmission electron microscope (CM120, Philips, Amsterdam, Netherlands).

### Western blotting

Cells were lysed in RIPA (Beyotime, P0013B) including 1% protease inhibitor cocktail (MedChem Express, HY-K0010), detached with a cell scraper, and centrifuged for 10 min at 12,000 × g. BCA protein assay kit (Beyotime, P0012) used to quantify protein concentrations. Cell lysates were boiled at 95°C for 10 min for conservation. Protein (20 μg) was electrophoresed in SDS-PAGE gels (Epizyme Biotech, PG112) using a Miniprotein III system (Bio-Rad, 1,658,033), transferred to PVDF membranes (Millipore, ISEQ00010) for 70 min, and then incubated in 5% skimmed milk in TBST (Servicebio, G0004) for 2 h at room temperature. PVDF were incubated with primary antibody overnight at 4°C. Then PVDF were washed with TBST three times, and incubated at room temperature for 1 h in peroxidase-conjugated goat anti-rabbit/mouse IgG secondary antibody. After washed with TBST, membranes were for chemiluminescence using the Super-sensitive ECL chemiluminescent substrate (Biosharp, BL520A).

The primary antibody are as follows: rabbit SQSTM1/p62 (1:10000; Abcam, ab109012), rabbit LC3B (1:2000; Abcam, ab192890), rabbit SNAP29 (1:1000; Abcam, ab181151), rabbit ATG5 (1:5000; Abcam, ab108327), rabbit STX17 (1:2000; Abcam, ab316119), rabbit VAMP8 (1:2000; Abcam, ab76021), rabbit Pan Kla (1:1000; PTM BIO, PTM-1401RM), mouse Pan Kac (1:1000; PTM BIO, PTM-101), mouse Flag (1:2000, Sigma-Aldrich, F1804), mouse ACTB/actin (1:5000; Affinity, T0022), rabbit TUBB/tubulin (1:5000; Affinity, AF7011), rabbit histone H3 (1:5000; Abcam, ab176842). ImageJ was used to calculate the band density. The relative expression levels of proteins were standardized using internal reference ACTB, TUBB or histone H3.

### Co-immunoprecipitation (co-IP)

Cells were lysed in Pierce™ IP Lysis Buffer (Thermo Fisher Scientific, 87,787) including 1% protease inhibitor cocktail (MedChem Express, HY-K0010), incubate on ice for 30 min and centrifuged for 20 min at 12,000 × g. Fully resuspend the Protein A/G Magnetic Beads (MedChem Express, HY-K0202) and transfer 35 μL into a 1.5 mL microcentrifuge tube. Resuspend the beads with 400 μL PBST (Servicebio, G2157) thoroughly, place the tube on a DynaMag™-2 magnetic rack (Thermo Fisher Scientific, 12321D) and discard the supernatant. Dilute the antibody with PBST to a final concentration of 10 μg/mL, add 400 μL of the diluted antibody to the washed magnetic beads, and incubate on a rotator at 4°C overnight. Separate the beads magnetically and resuspend the beads with 400 μL PBST. Add 400 μL prepared antigen sample to the antibody-magnetic bead complex, fully resuspend and incubate on a rotator at 4°C for 4 h. Separate the beads magnetically and resuspend the beads with 400 μL PBST. Add 25–50 μL of 1× SDS-PAGE Loading Buffer (Beyotime, P0015) to the beads and heat at 95°C for 10 min. Separate the beads magnetically, collect the supernatant for western blotting.

### Single-cell analysis

The single-cell transcriptomic dataset GSE214607 was downloaded from the Gene Expression Omnibus database. This dataset contains villous tissues from 5 normal pregnancies and 3 URPL cases. Raw expression matrices were processed and analyzed using the Seurat package in R (v4.5.1). Cells expressing fewer than 200 genes or more than 6000 genes, and cells with >10% mitochondrial gene expression were excluded. After quality control, data normalization and identification of highly variable genes were performed. Dimensionality reduction was achieved via principal component analysis and uniform manifold approximation and projection (UMAP). Cell clustering was conducted based on nearest-neighbor graphs, and major cell subtypes were annotated using canonical marker genes. Extravillous trophoblasts (EVT), cytotrophoblasts (CTB), and syncytiotrophoblasts (STB) were extracted from the integrated dataset for further analysis. Differentially expressed genes (DEGs) between URPL and control trophoblasts were identified with the Wilcoxon rank-sum test and visualization was performed using violin plots.

### 4D-FastDIA quantitative proteomics

HTR-8/SVneo cells were induced cellular senescence using H_2_O_2_, with or without exogenous IL33 supplementation (three biological replicates per group). After protein extraction and trypsin digestion, the sample were submitted to PTM BIO Co.Ltd. (Hangzhou, Zhejiang, China) for liquid chromatography-tandem mass spectrometry (LC-MS/MS) analysis. The DIA data were processed using DIA-NN search engine (v.1.8). Fisher’s exact test was used to analyze the significance of functional enrichment of differentially expressed proteins (using the identified protein as the background). Functional terms with Fold enrichment > 1.5 and P value < 0.05 were considered as significant. For further hierarchical clustering based on differentially expressed protein functional classification (GO, KEGG pathway, Reactome and WikiPathways). We first collated all the categories obtained after enrichment along with their P values, and then filtered for those categories which were at least enriched in one of the clusters with P value < 0.05. This filtered P value matrix was transformed by the function ×=−log10 (P value). These p values were then clustered by one-way hierarchical clustering (Euclidean distance, average linkage clustering) in Genesis. Cluster membership was visualized by a heat map using the “Heatmap” function from the “ComplexHeatmap” R-package.

### 4D fast DIA L-lactylation qualitative proteomics

After protein extraction and trypsin digestion, HTR-8/SVneo cells were submitted to PTM BIO Co.Ltd. (Hangzhou, Zhejiang, China) for affinity enrichment and LC-MS/MS analysis. The DIA data were processed using Spectronaut (v.18) software. Trypsin/P was specified as cleavage enzyme allowing up to 4 missing cleavages. Carbamidomethyl on Cys was specified as fixed modification. Acetylation on protein N-terminal, oxidation on Met and L-lactylation were specified as variable modifications. False discovery rate of protein, peptide and PSM was adjusted to <1%. Fisher’s exact test was used to analyze the significance of functional enrichment of identified proteins (using all proteins in the species database as the background). Functional terms with Fold enrichment > 1.5 and P value < 0.05 were considered as significant.

### Integration analysis of the protein–protein interaction (PPI) Network

The STRING database (http://string-db.org, accessed on 10 December 2024) was used for protein-protein interaction network prediction.

### Transfection

The SNAP29^K169R^ mutant plasmid was constructed by OBiO Technology Co., Ltd. (Shanghai, China). The SNAP29 wild-type overexpression plasmid (SNAP29 WT) was used as a control. The SNAP29-silencing plasmid (si-*SNAP29*) was also generated by OBiO Technology. si-*SNAP29* was transfected into HTR-8/SVneo and JAR cells to achieve SNAP29 knockdown. An empty vector with no target gene was used as a negative control. Transfections were performed using Lipofectamine 3000 (Invitrogen, 2,533,486) according to the manufacturer’s protocol. Cells were lysed 48 hours after transfection for subsequent analyses.

### Statistical analysis

Data were presented as mean ± standard error of the mean (Mean ± SEM). Normality of the data was tested with the Shapiro-Wilk’s test. Student’s t-test was used to analyze between two groups, and one-way analysis of variance (ANOVA) test was conducted among multiple groups. Two-tailed Mann Whitney test was used for non-normally distributed data. A statistically significant difference was considered at *p* < 0.05. All analyses were conducted with SPSS 26.0.

## Supplementary Material

Supplementary_Material_R3.docx

## Data Availability

The data that support the findings of this study are available from the corresponding author upon reasonable request.
