# Supplementary material for: Trophoblast aging driven by IL33 deficiency elevates recurrent pregnancy loss risk through SNAP29 lactylation-mediated autophagy impairment
Source: Autophagy. 2026 Apr 22;22(7):1679–97. doi: 10.1080/15548627.2026.2659946 (PMC13285577; doi:10.1080/15548627.2026.2659946)
Supplement: Supplementary_Material_R3.docx [file KAUP_A_2659946_SM0760.docx]

**Table S1.** Primer sequences used for qPCR in human cells.

| Gene | Sequence |
| --- | --- |
| *ACTB*  *IL6*  *IL8*  *CCL2*  *CDKN2A*  *CDKN1A*  *TP53*  *ATG5*  *MAP1LC3B*  *SQSTM1*  *LAMP1*  *LAMP2*  *STX3*  *VAMP8* | Forward: GCCGACAGGATGCAGAAGGAGATCA  Reverse: AAGCATTTGCGGTGGACGATGGA  Forward: ACTCACCTCTTCAGAACGAATTG  Reverse: CCATCTTTGGAAGGTTCAGGTTG  Forward: TTTTGCCAAGGAGTGCTAAAGA  Reverse: AACCCTCTGCACCCAGTTTTC  Forward: GAAAGTCTCTGCCGCCCTT  Reverse: GGTGACTGGGGCATTGATTG  Forward: GATCCAGGTGGGTAGAAGGTC  Reverse: CCCCTGCAAACTTCGTCCT  Forward: TGTCCGTCAGAACCCATGC  Reverse: AAAGTCGAAGTTCCATCGCTC  Forward: CAGCACATGACGGAGGTTGT  Reverse: TCATCCAAATACTCCACACGC  Forward: AAAGATGTGCTTCGAGATGTGT  Reverse: CACTTTGTCAGTTACCAACGTCA  Forward: GATGTCCGACTTATTCGAGAGC  Reverse: TTGAGCTGTAAGCGCCTTCTA  Forward: GCACCCCAATGTGATCTGC  Reverse: CGCTACACAAGTCGTAGTCTGG  Forward: TCTCAGTGAACTACGACACCA  Reverse: AGTGTATGTCCTCTTCCAAAAGC  Forward: GAAAATGCCACTTGCCTTTATGC  Reverse: AGGAAAAGCCAGGTCCGAAC  Forward: CGGCTTTTATGGACGAGTTCT  Reverse: CTGCCGATGACCTGACCTC  Forward: TGTGCGGAACCTGCAAAGT  Reverse: CTTCTGCGATGTCGTCTTGAA |

**Table S2.** Primer sequences used for qPCR in mouse tissues.

| Gene | Sequence |
| --- | --- |
| *Actb*  *Il6*  *Cxcl1*  *Tnf*  *Il1b*  *Tgfb1*  *Ppargc1a*  *Opa1*  *Mfn2*  *Dnm1l*  *Mff* | Forward: GTGACGTTGACATCCGTAAAGA  Reverse: GCCGGACTCATCGTACTCC  Forward: CTGCAAGAGACTTCCATCCAG  Reverse: AGTGGTATAGACAGGTCTGTTGG  Forward: ACTGCACCCAAACCGAAGTC  Reverse: TGGGGACACCTTTTAGCATCTT  Forward: CAGGCGGTGCCTATGTCTC  Reverse: CGATCACCCCGAAGTTCAGTAG  Forward: GAAATGCCACCTTTTGACAGTG  Reverse: TGGATGCTCTCATCAGGACAG  Forward: CCACCTGCAAGACCATCGAC  Reverse: CTGGCGAGCCTTAGTTTGGAC  Forward: TATGGAGTGACATAGAGTGTGCT  Reverse: GTCGCTACACCACTTCAATCC  Forward: TGGAAAATGGTTCGAGAGTCAG  Reverse: CATTCCGTCTCTAGGTTAAAGCG  Forward: AGAACTGGACCCGGTTACCA  Reverse: CACTTCGCTGATACCCCTGA  Forward: CCTCAGATCGTCGTAGTGGGA  Reverse: GTTCCTCTGGGAAGAAGGTCC  Forward: ATGCCAGTGTGATAATGCAAGT  Reverse: CTCGGCTCTCTTCGCTTTG |

**
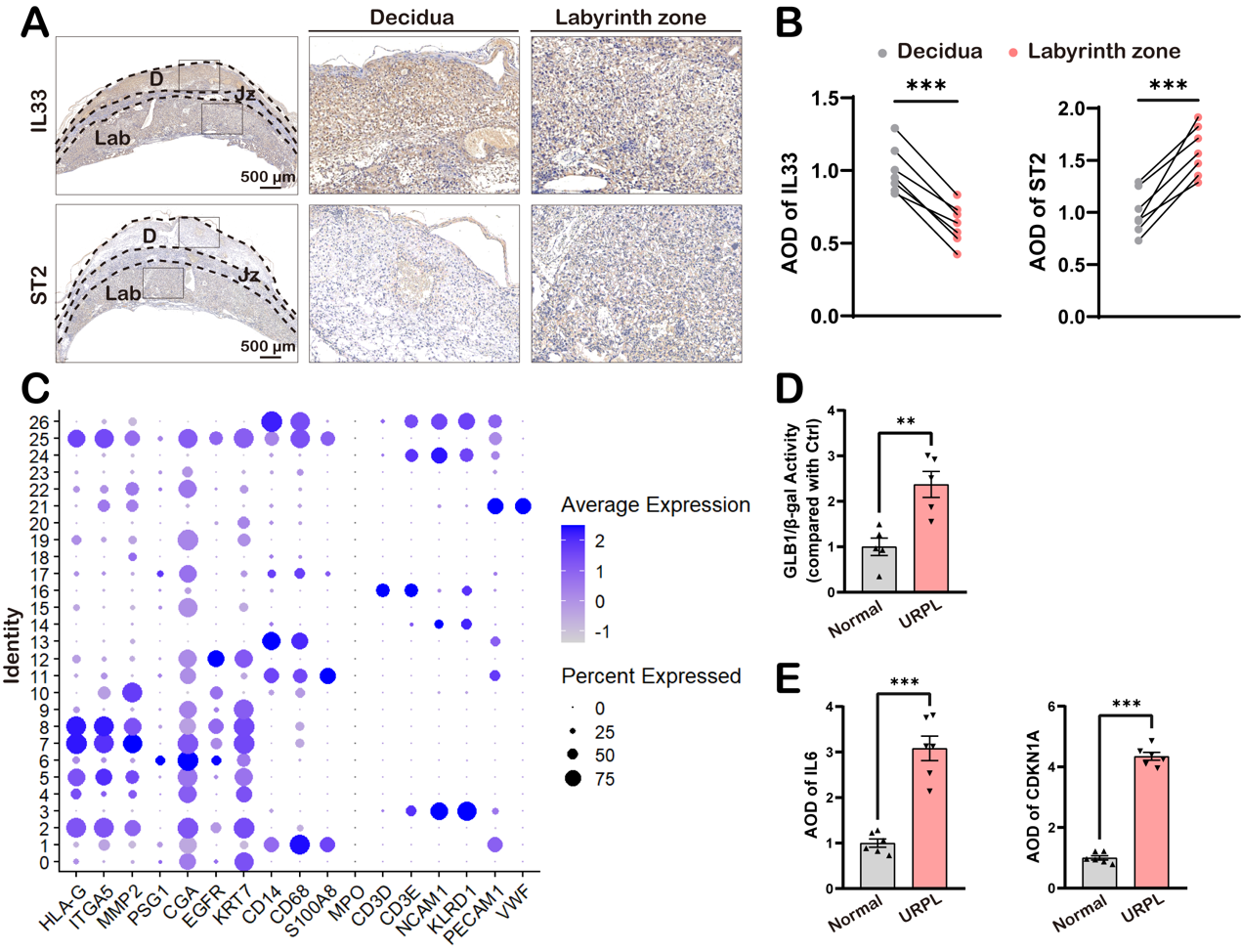
Figure S1.** IL33 deficiency induces trophoblast senescence and leads to pregnancy loss. (**A**) The expression of IL33 and ST2 was analyzed by immunohistochemistry staining in WT mice (n = 6). Scale bar: 500 μm. (**B**) Quantification of immunohistochemistry results of IL33 and ST2. (**C**) The expression of marker genes for the major cell types. (**D**) The activity of GLB1/β-galactosidase staining in normal and URPL villous tissues at 7-9 weeks of gestation (n = 6 per group). (**E**) Quantification of immunohistochemistry results of IL6 and CDKN1A in normal and URPL villi (n = 6 per group). The immunohistochemistry staining was quantified with integrated optical density value. Statistical data were presented as mean ± SEM. ***p<0.01, ***p<0.001*, using two-tailed Student’s t-test.


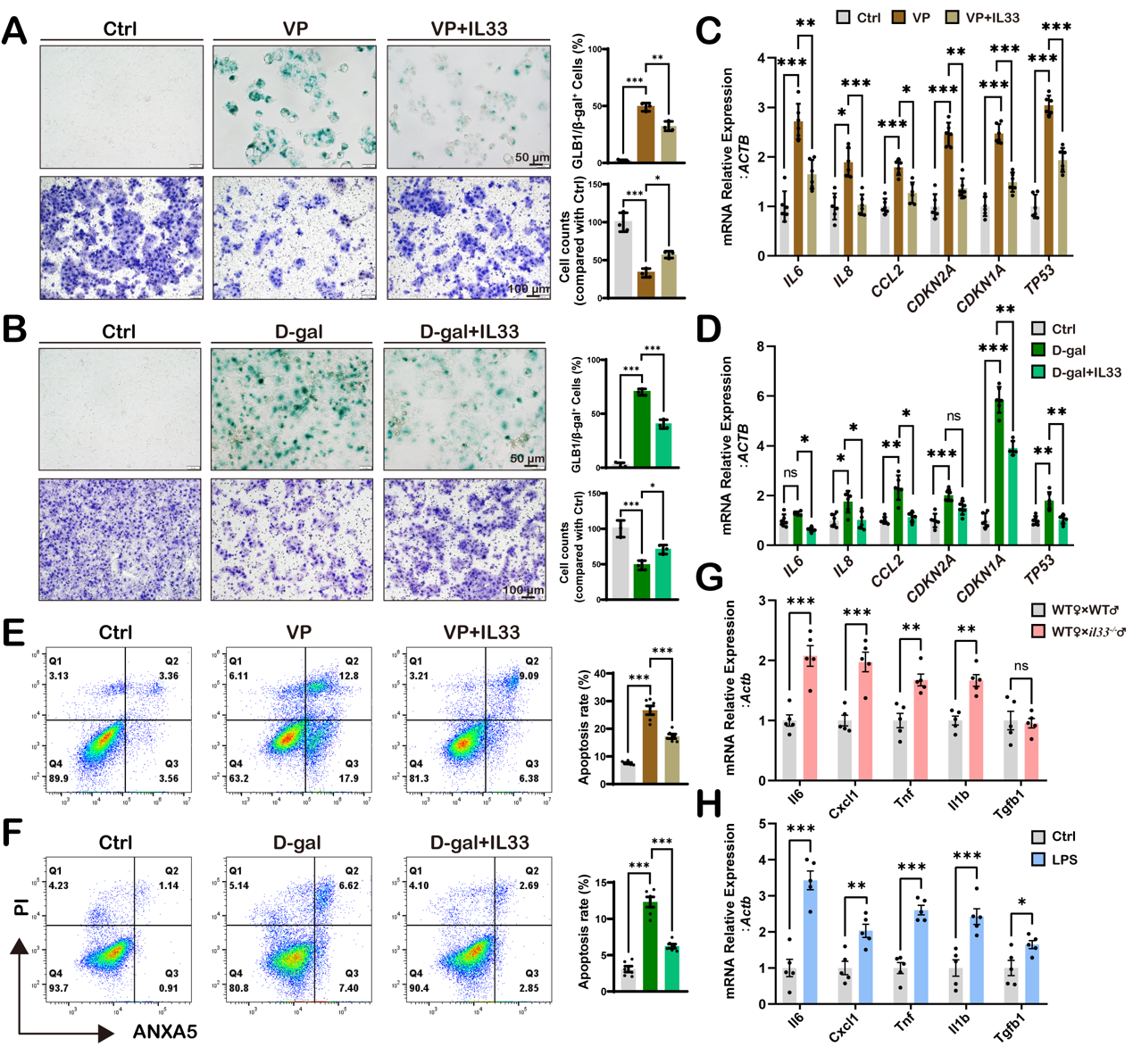


**Figure S2.** IL33 rescued the impaired function of senescent JAR cells. (**A and B**) GLB1/β-galactosidase staining and Matrigel invasion assay was performed in JAR cell senescence models treated with or without IL33 (10 ng/mL). Scale bar: 50 μm and 100 μm. (**C and D**) Relative mRNA expression levels of *IL6*, *IL8*, *CCL2*, *CDKN2A*, *CDKN1A* and *TP53* in JAR cell senescence models treated with or without IL33 (10 ng/mL). (**E and F**) The apoptosis rate was detected by the flow cytometry assay in JAR cell senescence models treated with or without IL33 (10 ng/mL). (**G and H**) Relative mRNA expression levels of *Il6*, *Cxcl1*, *Tnf*, *Il1b* and *Tgfb1* in mouse placental tissues (n = 5 per group). All data were generated using ImageJ. Statistical data were presented as mean ± SEM. **p<0.05, **p<0.01, ***p<0.001, ns, no significant difference*, using two-tailed Student’s t-test (G, H) and one-way ANOVA test (A-F).


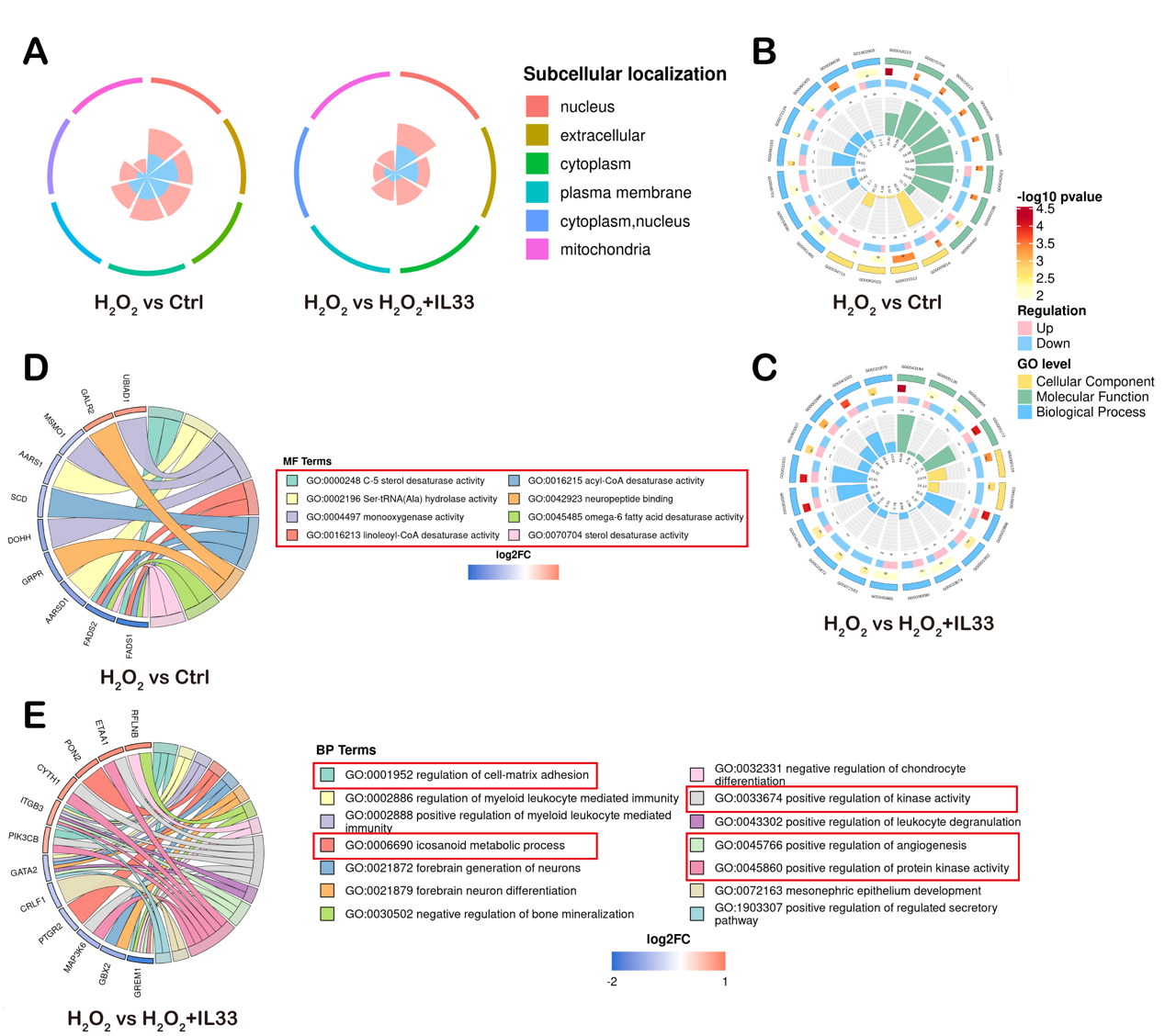


**Figure S3.** Characteristics of protein expression changes of H₂O₂-induced senescent HTR-8/SVneo cells treated with or without IL33. (**A**) Subcellular localization of differentially expressed proteins (DEPs) in H₂O₂-induced senescent HTR-8/SVneo cells, with or without IL33 supplementation (10 ng/mL). (**B and C**) GO enrichment analysis of DEPs across Cellular Component (CC), Molecular Function (MF), and Biological Process (BP) categories. Color bars indicate -log_10_(P value), and the inner ring represents the direction of regulation (up- or downregulated). (**D and E**) Chord plots of enriched MF terms (D) in H₂O₂ vs Ctrl and BP terms (E) in H₂O₂ vs H₂O₂+IL33 groups.


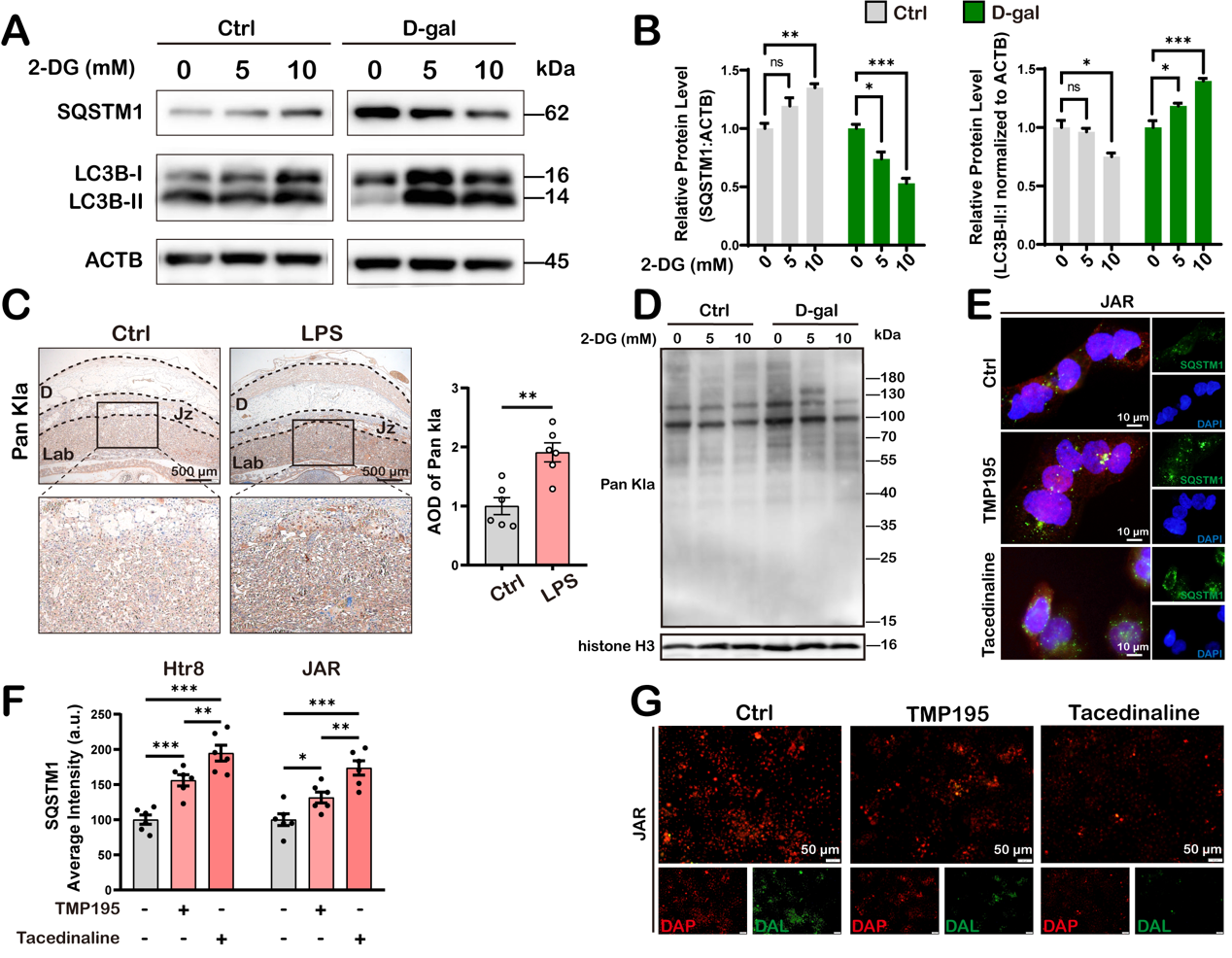


**Figure S4.** Senescence-induced glycolysis and lactate accumulation impair autophagy in trophoblasts. (**A and B**) Western blot for SQSTM1 or LC3B-II:I in normal and senescent HTR-8/SVneo cells treated with different concentration of 2-DG (0, 5, 10 mM for 48 h). Relative expression levels of proteins were standardized using internal reference ACTB. (**C**) The expression of lactylation level was analyzed by immunohistochemistry staining in control and LPS-treated mice (n = 6 per group). Scale bar: 500 μm. (**D**) Western blot for lactylation level in normal and senescent HTR-8/SVneo cells treated with different concentration of 2-DG (0, 5, 10 mM for 48 h). Relative expression levels of proteins were standardized using internal reference histone H3. (**E and F**) The expression of SQSTM1 was detected by immunofluorescence in HTR-8/SVneo and JAR cells treated with tacedinaline (5 μM，48 h) or TMP195 (5 μM，48 h). Scale bar: 10 μm. (**G**) The level of autophagy was detected by the DAL/DAP fluorescence probes in JAR cells treated with tacedinaline (5 μM，48 h) or TMP195 (5 μM，48 h). Scale bar: 50 μm. Immunofluorescence staining results was quantified with average intensity. Statistical data were presented as mean ± SEM. **p<0.05, **p<0.01, ***p<0.001, ns, no significant difference*, using two-tailed Student’s t-test (C) and one-way ANOVA test (B, F).


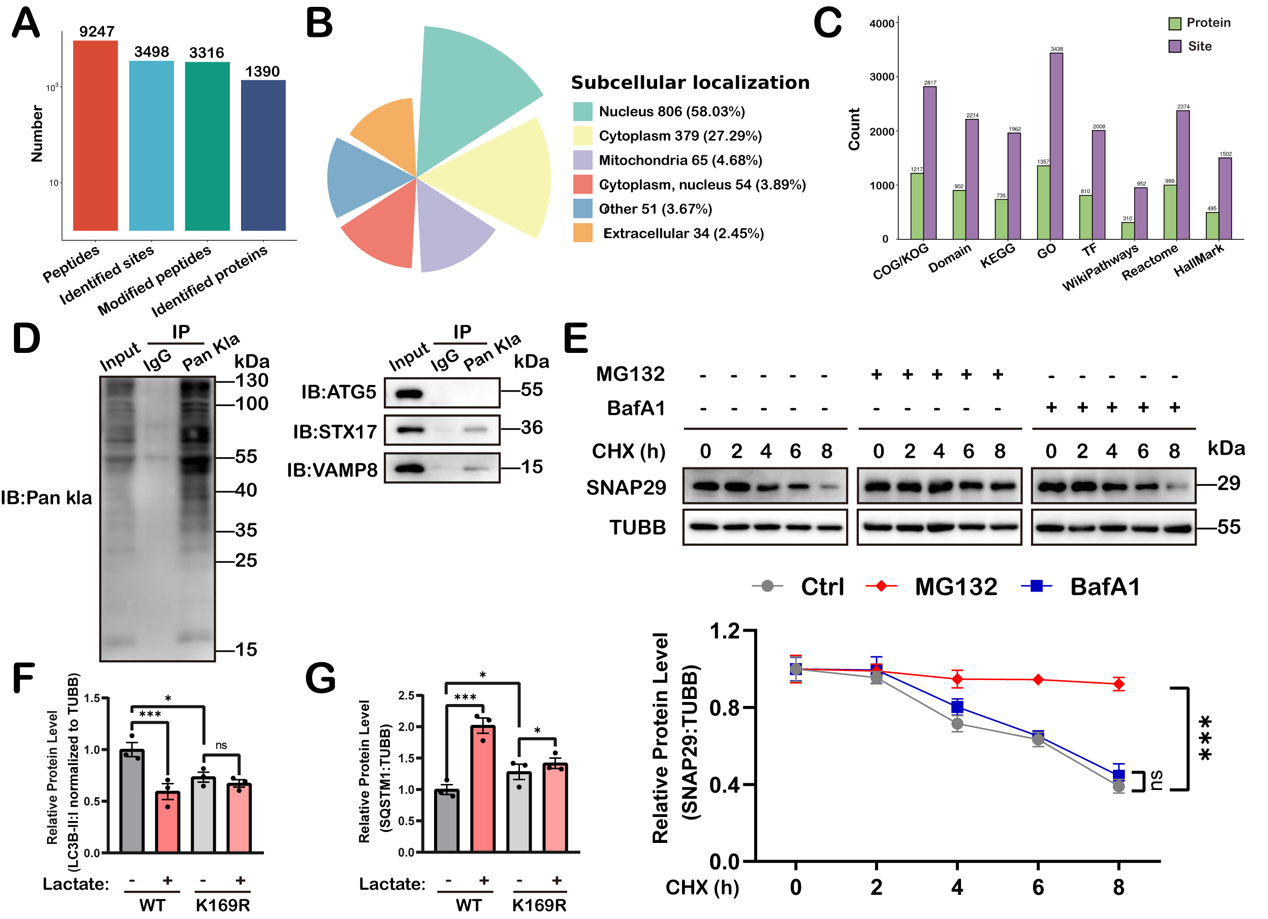


**Figure S5.** Lactylation of SNAP29 suppresses autophagy and compromises trophoblast invasive capacity. (**A**) Proteins and modification sites identified by lactylation proteomics. (**B**) Subcellular localization of lactylated proteins. (**C**) Functional enrichment analysis (KEGG, GO, Reactome, etc.) of identified lactylated proteins and their modification sites. (**D**) Co-immunoprecipitation was performed using pan lactylation antibodies in HTR-8/SVneo cells. (**E**) Western blot for SNAP29 in HTR-8/SVneo cells treated with CHX (30 μg/mL, 0–8 h) in the presence or absence of MG132 (10 µM) or bafilomycin A_1_ (BafA1, 100 nM). Relative expression levels of proteins were standardized using internal reference TUBB. (**F and G**) Western blot for SQSTM1 and LC3B-II:I in WT vs. K169R HTR-8/SVneo cells after lactate treatment (20 mM, 48 h). Relative expression levels of proteins were standardized using internal reference TUBB. Statistical data were presented as mean ± SEM. **p<0.05, ***p<0.001, ns, no significant difference*, using one-way ANOVA test.


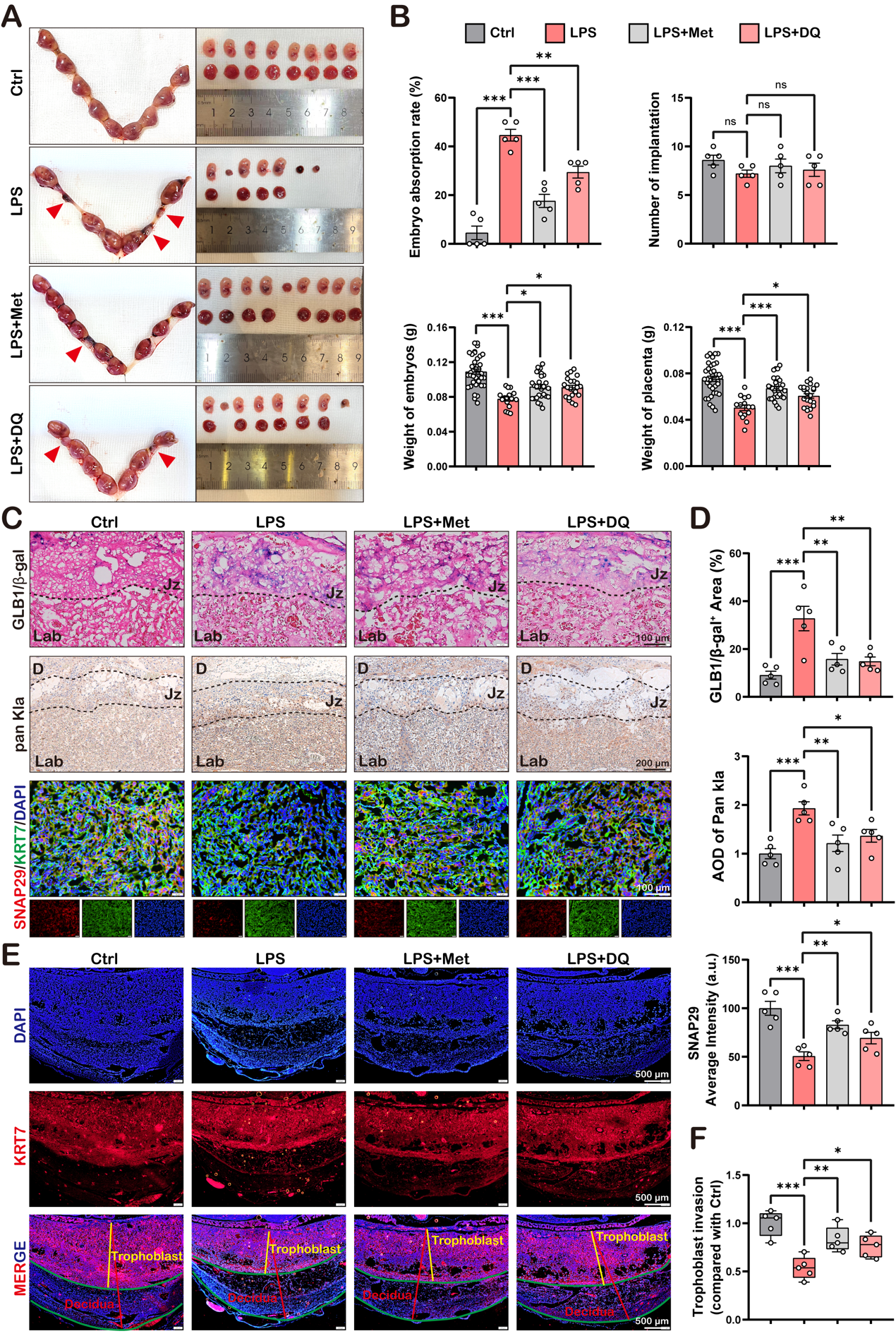


**Figure S6.** Anti-senescence interventions alleviate placental dysfunction and rescue the pregnancy loss cause by LPS. (**A and B**) The pregnancy outcomes at gestational day 13.5 were assessed in the control (n = 5), LPS-treated (n = 5), LPS + metformin (n = 5), and LPS + DQ (n = 5) groups. Embryo resorption rate, number of blastocyst implantation, weight of placenta and embryo were observed in mice. (**C and D**) Placental senescence was assessed by GLB1/β-galactosidase staining, lactylation levels were examined by immunohistochemistry, and SNAP29 expression was evaluated by immunofluorescence (n = 5 per group). The immunohistochemistry staining was quantified with average optical density (AOD) and immunofluorescence staining was quantified with average intensity. Scale bar: 100 μm and 200 μm. (**E and F**) The depth of trophoblast infiltration in the placenta was assessed by immunofluorescence (n = 5 per group). The ratio of trophoblast infiltration (yellow line) to the depth of the entire uterus (red line) was analyzed. Scale bar: 500 μm. All data were generated using ImageJ. Statistical data were presented as mean ± SEM. **p<0.05, **p<0.01, ***p<0.001, ns, no significant difference,* using one-way ANOVA test.


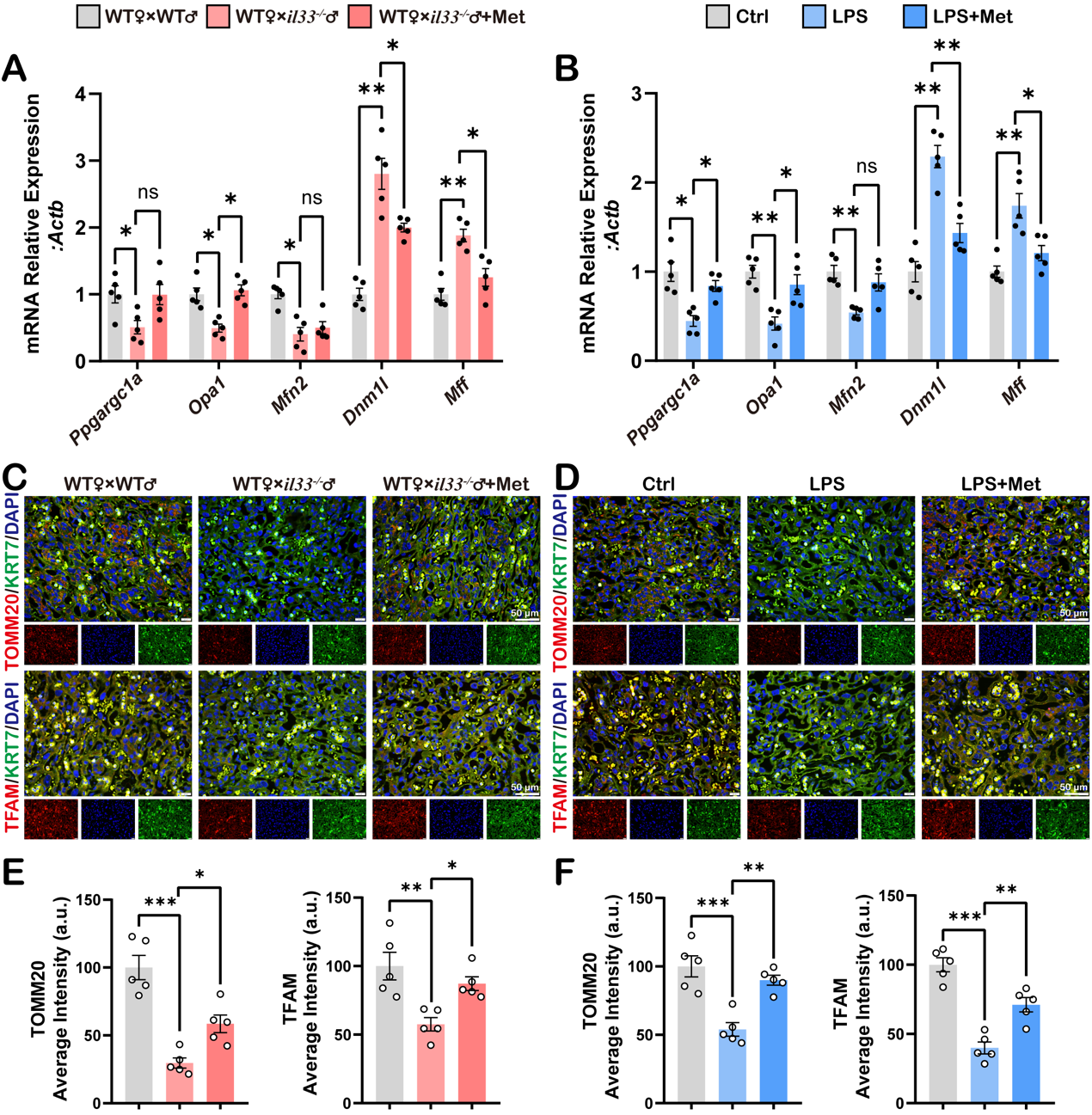


**Figure S7.** Metformin alleviates senescence-associated mitochondrial alterations in mouse placentas. (**A and B**) Relative mRNA expression levels of *Ppargc1a*, *Opa1*, *Mfn2*, *Dnm1l* and *Mff* in mouse placental tissues (n = 5 per group). (**C and D**) TOMM20 and TFAM expression were evaluated by immunofluorescence (n = 5 per group). Scale bar: 50 μm. (**E and F**) Immunofluorescence staining was quantified with average intensity. All data were generated using ImageJ. Statistical data were presented as mean ± SEM. **p<0.05, **p<0.01, ***p<0.001, ns, no significant difference,* using one-way ANOVA test.
